# The interaction between ΔNp63α and TAp63α, mediated by miR-205-5p, inhibits the migration of lung adenocarcinoma cells

**DOI:** 10.1038/s41598-025-95206-4

**Published:** 2025-04-03

**Authors:** Samaneh Qobadi-Nasr, Mohammad Hossein Pourgholami, Seyed Javad Mowla

**Affiliations:** 1 Department of Molecular Genetics, Faculty of Biological Sciences, Tarbiat Modares University, Tehran, 14115- 111 Iran; 2 Department of Physiology, Faculty of Medical Sciences, Tarbiat Modares University, Tehran, 14115-111 Iran

**Keywords:** Lung cancer, ΔNp63, TAp63, miR-205, DICER, Migration, Cancer, Genetics, Molecular biology

## Abstract

Lung cancer is a highly lethal disease worldwide, resulting from a combination of genetic, epigenetic, and environmental factors. The amplification of specific chromosomal regions is a hallmark of cancer progression; for instance, the 3q region of chromosome 3 is notably amplified in lung cancer, contributing to early tumor development. *TP63*, a member of the p53 family, is located in the 3q region. The presence of two distinct sets of *TP63* isoforms (ΔNp63 and TAp63) complicates its functional role. Furthermore, miR-205-5p, a crucial player in cancer progression, has a predicted target site in the 5’-untranslated region (5’-UTR) of TAp63 transcripts. To investigate a potential correlation between miR-205-5p and the ΔNp63 and TAp63 isoforms, we conducted an in silico study followed by experimental validations on clinical tissue samples. We found a significant positive correlation between the expression of miR-205-5p and both isoforms of *TP63* in lung adenocarcinoma (LUAD) datasets. The correlation between ΔNp63 and miR-205-5p was further confirmed in tissue samples from LUAD patients. Subsequently, we overexpressed ΔNp63α in lung adenocarcinoma cell lines and observed an upregulation of miR-205-5p, TAp63α, and DICER in the A549 cell line. Overexpression of ΔNp63α also inhibited the migration of A549 cells by reducing epithelial-mesenchymal transition (EMT) markers and increasing mesenchymal-epithelial transition (MET) markers. We conducted a luciferase assay to investigate the direct interaction between miR-205-5p and the 5’-UTR of TAp63 and observed a positive association. Overexpression of miR-205-5p in the A549 cell line led to the upregulation of TAp63α and DICER. Additionally, we found a reduction in migration following miR-205-5p transfection. Based on these results, it appears that there is a ΔNp63α/miR-205-5p/TAp63α/DICER axis involved in the regulation of migration in lung adenocarcinoma, which is cell-specific.

**Supplementary Information:**

The online version contains supplementary material available at 10.1038/s41598-025-95206-4.

## Introduction

Lung cancer is a prevalent form of cancer, accounting for 1.8 million deaths and 2.2 million diagnosed cases in 2020. It is the second leading cause of cancer-related deaths among women and the primary cause among men worldwide^1^. Lung cancer is a complex disease resulting from a combination of genetic and epigenetic alterations. Factors such as single nucleotide mutations, changes in the number of chromosome copies, and deletions or amplifications of specific genomic regions contribute to the progression of lung cancer. One of the initial events in the development of lung cancer is the amplification of the 3q chromosome^2^.

Tumor protein 63 (TP63) is a transcription factor predominantly found in epithelial cells. Its gene is located in the 3q28 amplification region. TP63 plays a crucial role in various biological processes, including differentiation, motility, stem cell maintenance, cell survival, and the epithelial-mesenchymal transition (EMT)^3^. The *TP63* gene features alternative promoters that regulate the transcription of two sets of isoforms. The promoter located upstream of exon 1 encodes the long isoforms (TAp63s), which contain a p53-like transcriptional activation domain. An alternative promoter situated in the intron downstream of exon 3 generates truncated isoforms (ΔNp63s) that lack the p53-like transcriptional activation domain. The primary transcript undergoes alternative splicing at the carboxyl terminus (-COOH), resulting in at least ten isoforms of the *TP63* gene (TAp63α/β/γ/δ/ε and ΔNp63α/β/γ/δ/ε)^4–7^.

TAp63s and ΔNp63s have distinct effects on carcinogenesis. TAp63s, which contain a transcriptional activation domain similar to that of p53, regulate the expression of p53 target genes, including *P21*, *BAX*, *PUMA*, and *LKB1*. Consequently, TAp63s typically function as tumor suppressors. Additionally, TAp63s activate genes that are independent of p53, such as *IAPP*, *SMARCD3*, and *DICER*. Initially, it was thought that ΔNp63s inhibit the expression of p53 and TAp63 target genes due to their lack of the N-terminal transcriptional activation domain, leading to the belief that they act as oncogenes. However, subsequent research has shown that ΔNp63s possess a truncated transcriptional activation domain (TAD) in their N-terminal region. These isoforms are capable of transcribing various genes, including *BRACHYURY*, *JAG1*, *DGCR8*, *HAGH*, *PERP*, *CASPASE-1*, *K14*, *BPAG1*, *MKP3*, and *HSP70*^4,8^.

It has been observed that the *TP63* gene is amplified in 88% of lung squamous carcinoma (LUSC) cases and in 11% of lung adenocarcinoma (LUAD) cases. The ΔNp63α variant is the most commonly expressed form of this gene. Furthermore, patients with non-small cell lung cancer (NSCLC) who exhibit amplification and overexpression of *TP63* tend to have a higher survival rate^9^. A study conducted by Iacono et al. demonstrated that altering the ΔNp63/TAp63 ratio significantly impacts tumor development more than merely increasing the expression of a single isoform^10^.

MicroRNAs (miRNAs) are small non-coding RNA molecules that play crucial roles in post-transcriptional gene regulation. Numerous studies have demonstrated that miRNAs can function as either oncogenes or tumor suppressors in various types of cancer^11^. MicroRNA-205 (miR-205) is a highly conserved miRNA found in epithelial tissues and can act as either an oncogene or a tumor suppressor, depending on the origin and stage of the cancer^12^.

In prostate and breast cancer, miR-205 inhibits tumor growth and migration while inducing apoptosis. Additionally, in breast cancer, miR-205 enhances radiosensitivity by targeting HER3 and ZEB factors^13,14^. Conversely, in endometrial cancer, miR-205 inhibits apoptosis, promotes cell proliferation by reducing PTEN levels, and facilitates EMT by activating the AKT pathway^15^. By targeting PTEN and stimulating AKT signaling, miR-205 promotes proliferation, migration, radioresistance, and invasion in nasopharyngeal cancer^16^. Furthermore, miR-205 is involved in angiogenesis and tumor progression in ovarian cancer^17^.

Multiple studies have demonstrated heterogeneity in miR-205 expression within tumors of the same type, particularly in non-small cell lung cancer and esophageal cancer. In this context, squamous cell carcinomas exhibit elevated levels of miR-205, while adenocarcinomas show reduced expression^18–20^. In summary, research highlights the dual function of miR-205, raising important questions regarding the mechanisms of its expression.

It has been demonstrated that ΔNp63α functions as a transcription factor for miR-205 in bladder and prostate cancers^13,21^. The miRcode database also predicted a target site for miR-205-5p in the 5’-UTR of TAp63 isoforms. Therefore, there appears to be a regulatory link among ΔNp63α, miR-205-5p, and TAp63 isoforms, which our study aimed to investigate. Initially, we conducted a computational analysis to establish the correlation between miR-205-5p and ΔNp63α, as well as between miR-205-5p and TAp63α in the LUAD and LUSC datasets. Afterward, we investigated the correlation between *TP63* isoforms and miR-205-5p in tissue samples of LUAD and LUSC. Subsequently, we assessed the effects of ΔNp63α upregulation on miR-205-5p and TAp63 expression, along with cellular processes such as apoptosis, cell cycle progression, proliferation, and migration. We employed a luciferase assay to explore the direct interaction between miR-205-5p and the 5’-UTR of TAp63. In the subsequent phase of our study, we examined the changes in cellular function resulting from the overexpression of miR-205-5p.

## Results

### In silico **analysis demonstrated a positive correlation between miR-205-5p and both TAp63α and ΔNp63α isoforms in LUAD**

**Fig. 1:**
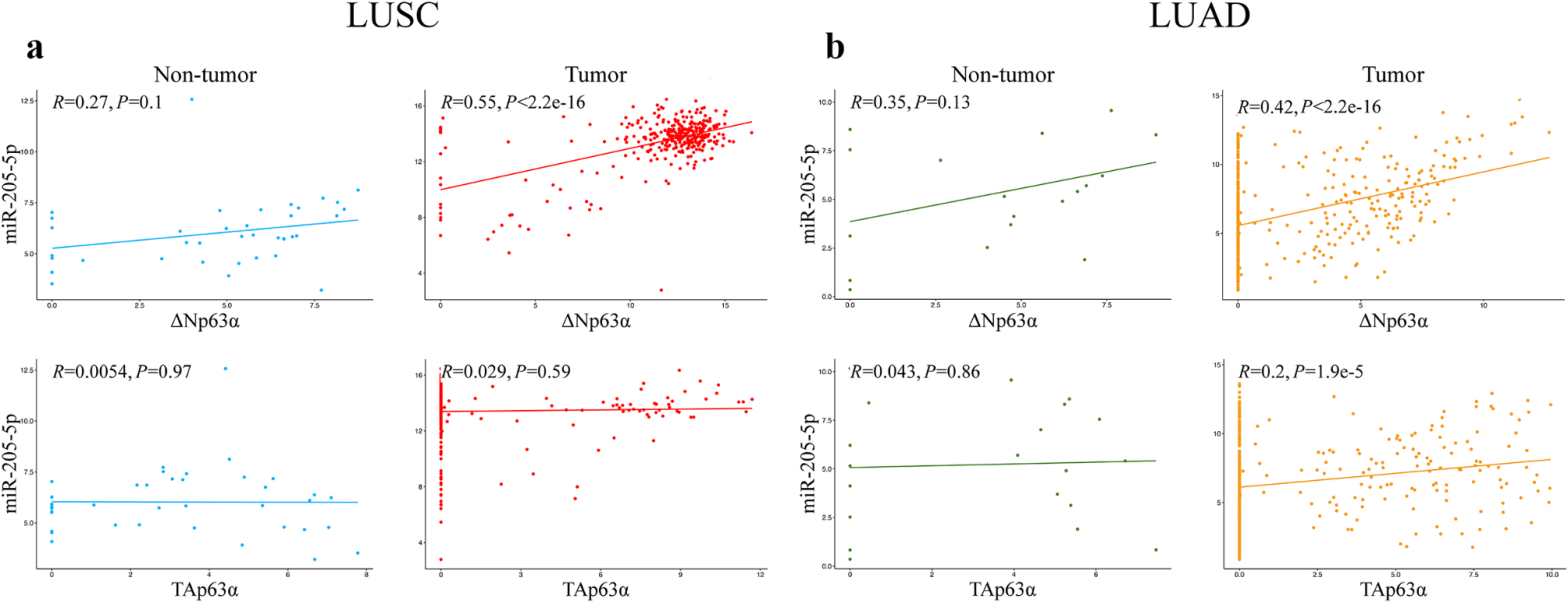
**Positive correlation between miR-205-5p and** ***TP63*** **isoforms in the LUAD dataset.** (**a**) In LUSC samples, the co-expression study showed a positive correlation between miR-205-5p and ∆Np63α (*P* < 2.2 × 10^–16^), but did not show any significant correlation between miR-205-5p and TAp63α expression (*P* = 0.59). (**b**) In LUAD samples, a positive correlation was observed between miR-205-5p and both TAp63α and ∆Np63α expression (*P* < 2.2 × 10^–16^ for miR-205-5p and ∆Np63α co-expression, *P* = 1.9 × 10^−5^ for miR-205-5p and TAp63α co-expression).

The co-expression study revealed a strong positive correlation between miR-205-5p and ΔNp63α expression in LUAD and LUSC samples (*P* < 2.2 × 10^–16^). However, no significant correlation was observed in non-tumor samples. The co-expression analysis of miR-205-5p and TAp63α demonstrated a substantial positive correlation in LUAD samples (*P* = 1.9 × 10^−5^). Nevertheless, this correlation was not statistically significant in LUSC and non-tumor samples (Fig. 1).

### **Correlation between the expression of miR-205-5p and*TP63*isoforms in tissue samples of LUSC and LUAD**

**Fig. 2:**
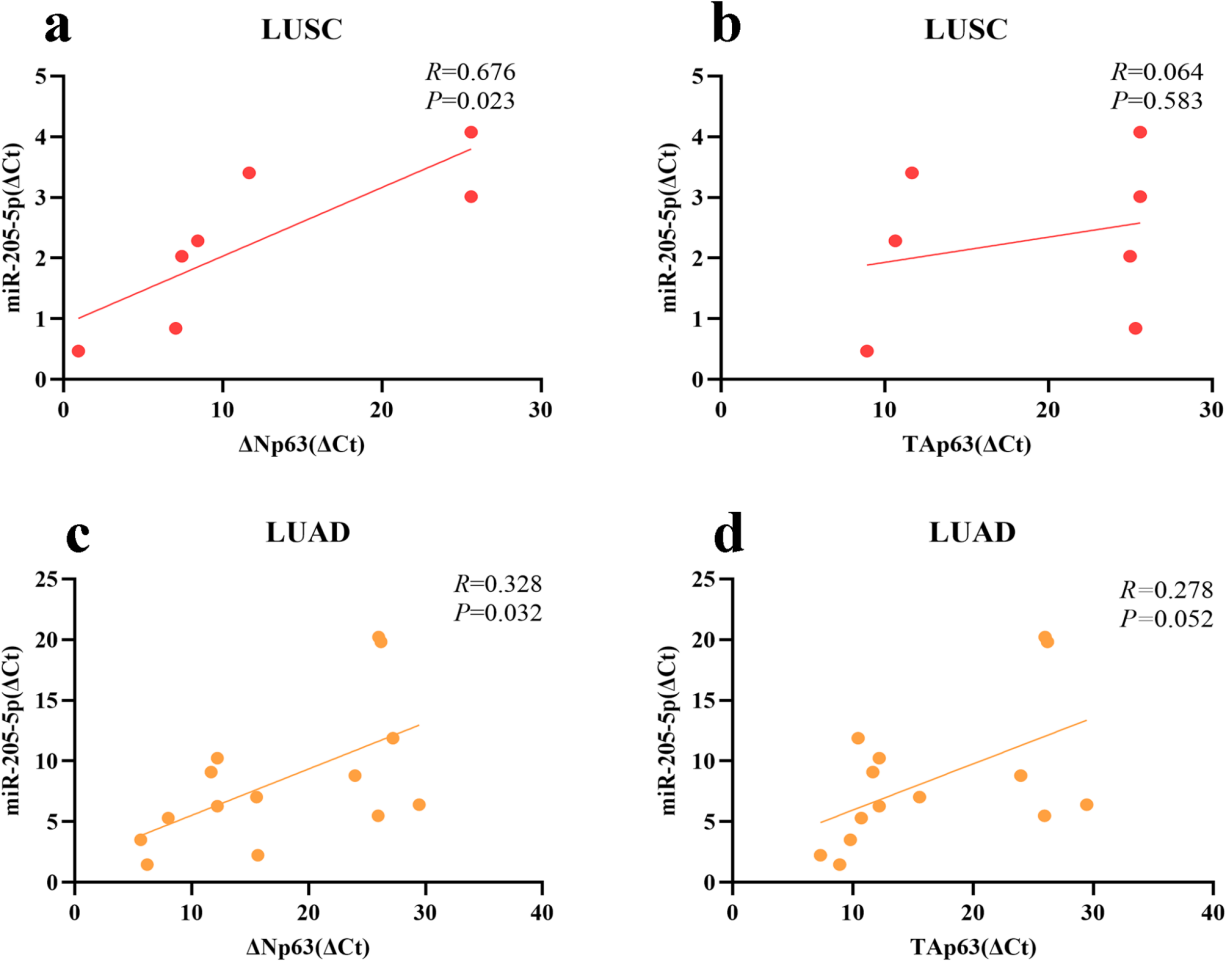
Positive correlation between miR-205-5p and ∆Np63 expression in tissue samples of LUAD and LUSC. (**a**,** c**) qRT-PCR results indicated a positive correlation between miR-205-5p and ∆Np63 expression in both LUSC and LUAD samples. (**b**,** d**) No significant correlation was observed between miR-205-5p and the TAp63 isoform in LUAD and LUSC samples.

In the 14 LUAD samples, ΔNp63 expression was detected in 3 samples, while TAp63 expression was observed in 5 samples. Additionally, 9 samples showed no expression of either isoform. Among the 7 LUSC samples, ΔNp63 expression was found in 4 samples, TAp63 expression was detected in 2 samples, and none of the isoforms were expressed in 3 samples. Quantitative reverse transcription polymerase chain reaction (qRT-PCR) analysis revealed a significant positive correlation between miR-205-5p and ΔNp63 in both LUSC and LUAD samples (*P* = 0.023 for LUSC and *P* = 0.032 for LUAD) (Fig. 2a, c). However, the correlation between miR-205-5p and TAp63 was not statistically significant in either LUSC or LUAD samples (Fig. 2b, d).

#### Overexpression of ΔNp63α increased the levels of miR-205-5p and TAp63 in A549 cells

**Fig. 3:**
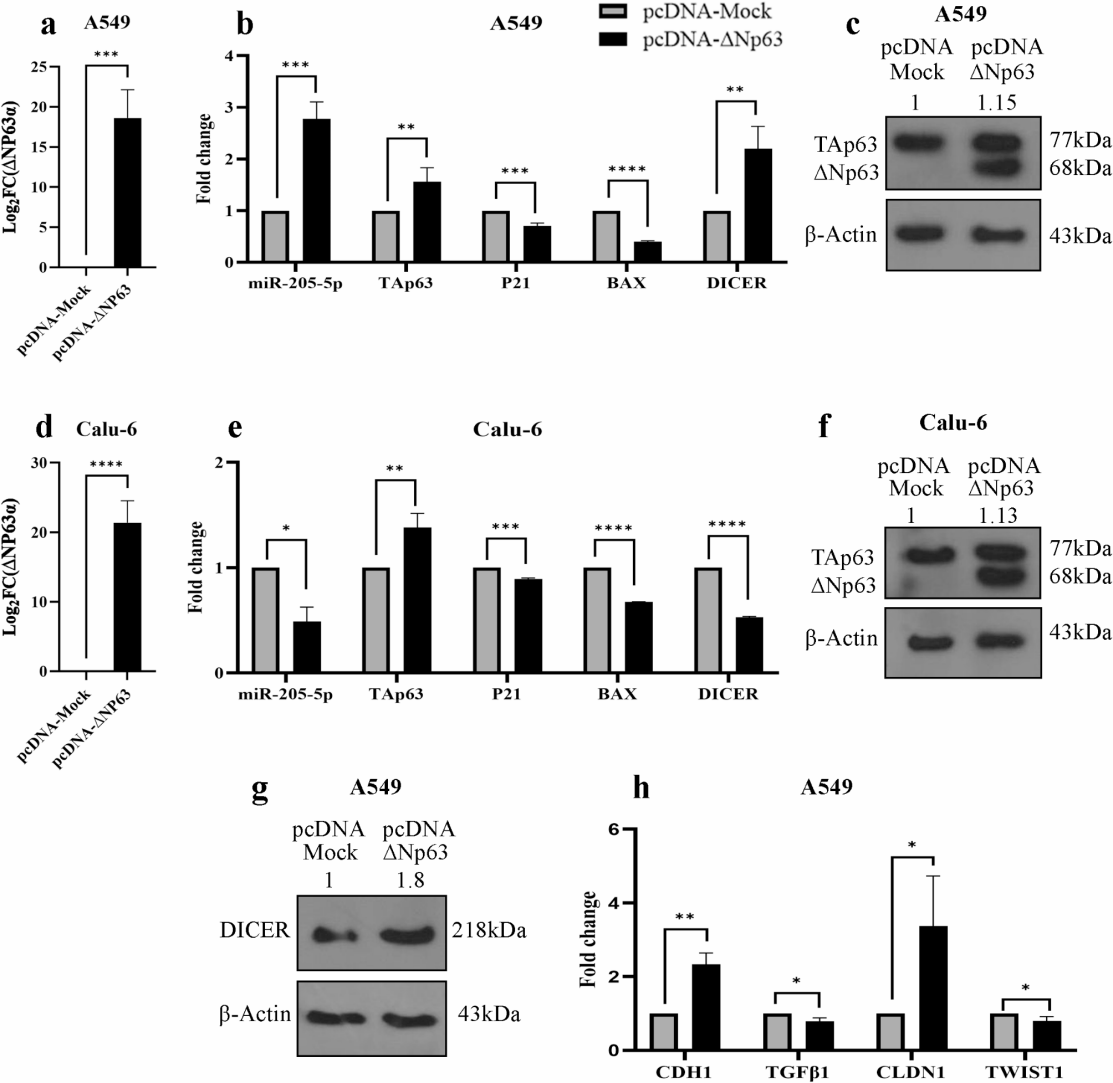
Overexpression of ΔNp63α enhanced miR-205-5p and TAp63 expression in the A549 cell line. (**a**,** d**) Overexpression of ΔNp63α in A549 and Calu-6 cells was confirmed by qRT-PCR. (**b**) Transfection of ΔNp63α in the A549 cell line resulted in significant upregulation of miR-205-5p (*P* = 0.0008) and TAp63 (*P* = 0.0083) expression. BAX (*P* < 0.0001) and P21 (*P* = 0.0008) levels decreased, while DICER expression increased considerably (*P* = 0.0029). (**e**) In Calu-6 cells, ΔNp63α overexpression reduced miR-205-5p (*P* = 0.0343), BAX (*P* < 0.0001), P21 (*P* = 0.0002), and DICER (*P* < 0.0001) expression, but TAp63 (*P* = 0.0029) was upregulated. (**c**,** f**) In both cell lines, an increase in TAp63 protein level following ΔNp63α overexpression was confirmed using western blot analysis. The western blot images were cropped from the original images, which are available in the supplementary file. An SDS-PAGE gel was run, including a molecular weight marker ,extracted protein from Calu-6 cells transfected with pcDNA-Mock, Calu-6 cells transfected with pcDNA-ΔNp63, A549 cells transfected with pcDNA-Mock, and A549 cells transfected with pcDNA-ΔNp63. After transferring the proteins from the SDS-PAGE gel to a PVDF membrane, the membrane was incubated with an anti-TP63 antibody. Following visualizing the bands, the membrane was washed and re-incubated with an anti-β-actin antibody, which was used as a loading control. (**g**) Western blot results indicated upregulation of DICER at the protein level in A549 cells transfected with pcDNA-ΔNp63. The western blot images were cropped from the original images, which are available in the supplementary file. An SDS-PAGE gel was run, including a molecular weight marker, extracted protein from A549 cells transfected with pcDNA-Mock, and extracted protein from A549 cells transfected with pcDNA-ΔNp63. After transferring the proteins from the SDS-PAGE gel to a PVDF membrane, the membrane was incubated with an anti-Dicer antibody. Following visualizing the bands,the membrane was washed and re-incubated with an anti-β-actin antibody (**h**) Expression of MET markers increased while EMT markers downregulated after ΔNp63α overexpression in A549. *P* value < 0.05: *, *P* value < 0.01: **, *P* value < 0.001: ***, *P* value < 0.0001: ****.

Following the transfection of pcDNA-∆Np63 into A549 and Calu-6 cells, RNA was extracted, and the expression of the target genes was quantified using qRT-PCR. The results from qRT-PCR and Western blot analyses indicated that the transfection of pcDNA-∆Np63 led to a significant overexpression of ΔNp63α in both the A549 (*P* = 0.0008) and Calu-6 (*P* < 0.0001) cell lines (Fig. 3a, c, d, f). We subsequently assessed the expression levels of miR-205-5p and TAp63 in both cell lines. The data revealed a significant increase in miR-205-5p (*P* = 0.0008) and TAp63 (*P* = 0.0083) expression in the A549 cell line following ΔNp63α overexpression (Fig. 3b). Western blot results confirmed the upregulation of TAp63 in this cell line (Fig. 3c). In contrast, the results for the Calu-6 cell line differed; ΔNp63α overexpression resulted in downregulation of miR-205-5p, while TAp63 levels were elevated at both the mRNA and protein levels (Fig. 3e, f). Given the upregulation of TAp63 in both cell lines, we examined its downstream targets, including *BAX*, *P21*, and *DICER*. The results indicated a decrease in BAX and P21 expression, along with an increase in DICER expression in the A549 cell line (Fig. 3b). Conversely, the Calu-6 cell line exhibited decreased expression of BAX, P21, and DICER (Fig. 3e). In the A549 cell line, the alteration in DICER expression correlated with the upregulation of TAp63, prompting us to use Western blotting to confirm this relationship at the protein level. The Western blotting results demonstrated a substantial increase in DICER expression at the protein level (Fig. 3g). We then investigated the expression of EMT and MET markers in A549 cells transfected with pcDNA-∆Np63. The qRT-PCR results showed a significant increase in CDH1 (*P* = 0.0039) and CLDN1 (*P* = 0.0129) expression as MET markers, while the expression of EMT markers, TGFβ1 (*P* = 0.0181) and TWIST1 (*P* = 0.0447), exhibited a decrease (Fig. 3h).

## Effects of ΔNp63α overexpression on apoptosis, proliferation, and cell migration

**Fig. 4:**
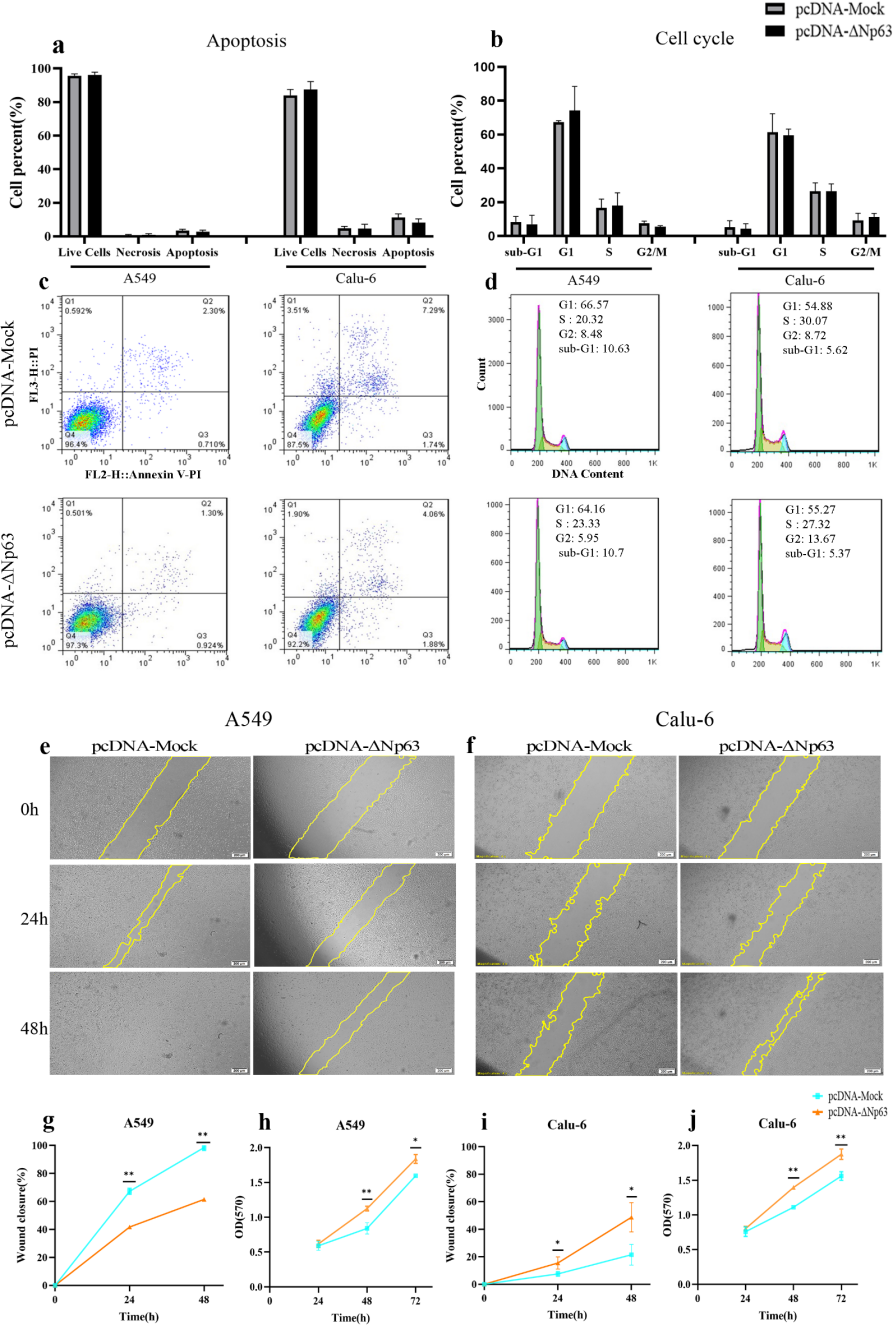
Effects of ∆Np63α overexpression on cell activities. (**a**,** b**,** c**,** d**) In both cell lines, ∆Np63α did not affect the apoptosis rate or cell cycle. (**e**,** g**) Overexpression of ∆Np63α significantly reduced migration in A549 cells (*P* = 0.0038 at 24 h, *P* = 0.0024 at 48 h). (**h**) ∆Np63α overexpression in the A549 cell line contributed to a considerable increase in proliferation rate at 48 (*P* = 0.0052) and 72 (*P* = 0.0378) hours after transfection. (**f**,** i**) In contrast to the observed results in the A549 cell line, ∆Np63α led to the induction of migration in the Calu-6 cell line (*P* = 0.0463 at 24 h, *P* = 0.0225 at 48 h). (**j**) The MTT result in the Calu-6 cell line was similar to A549 and demonstrated an increase in proliferation rate (*P* = 0.0074 at 48 h, *P* = 0.0061 at 72 h). *P* value < 0.05: *, *P* value < 0.01: **.

The results of flow cytometry indicated that the overexpression of ΔNp63α did not significantly affect the apoptosis rate or cell cycle in either cell line (Fig. 4a, b, c, d). Based on these findings, it appears that ΔNp63α is not involved in apoptosis or cell cycle pathways in adenocarcinoma cell lines. The results of the wound-healing assay were particularly noteworthy. As shown in Fig. 4, the overexpression of ΔNp63α led to a significant decrease in the migration of A549 cells (*P* = 0.0038 at 24 h, *P* = 0.0024 at 48 h). A549 cells transfected with pcDNA-Mock completely closed the wound after 48 h, while wounds in pcDNA-ΔNp63 transfected cells remained open (Fig. 4e, g). In contrast, Calu-6 cell lines responded differently to ΔNp63α overexpression in wound-healing assays. In pcDNA-ΔNp63 transfected Calu-6 cells, the overexpression of ΔNp63α significantly increased migration at various time points (*P* = 0.0463 at 24 h, *P* = 0.0225 at 48 h) (Fig. 4f, i). To investigate the effect of ΔNp63α on proliferation rates, we conducted an MTT assay. The results of the assay were identical for both cell lines, indicating that ΔNp63α overexpression led to a substantial increase in cell proliferation at 48 and 72 h post-transfection, although no significant change was observed at 24 h (Fig. 4h, j).

### **TAp63 is a direct target of miR-205-5p**,** and the overexpression of miR-205-5p in the A549 cell line leads to the upregulation of both TAp63 and DICER**

**Fig. 5:**
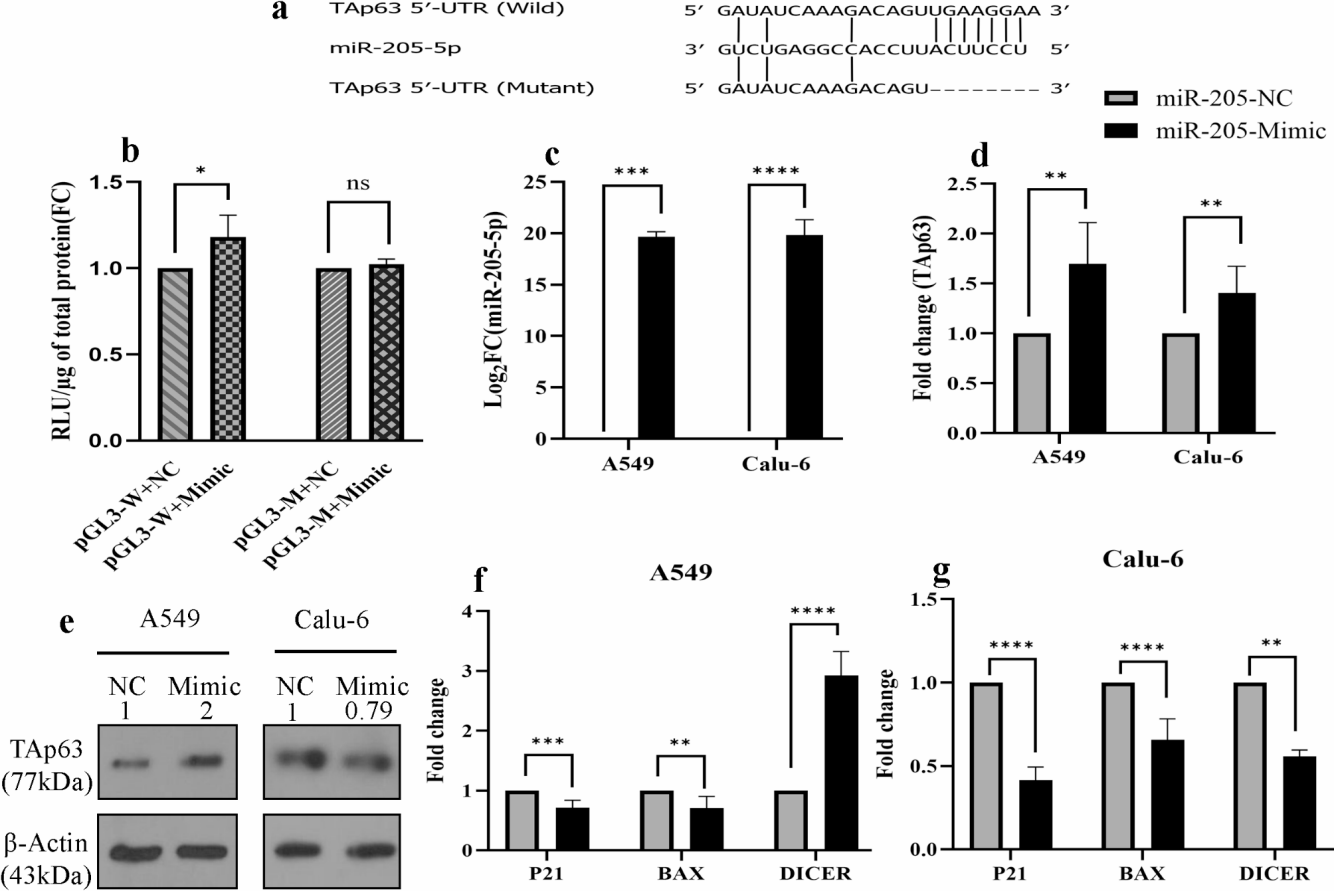
TAp63 was a direct target for miR-205-5p. (**a**) The predicted target site of miR-205-5p on TAp63 5’-UTR. (**b**) miR-205-5p and TAp63 5’-UTR exhibited direct interaction that enhanced luciferase activity. (**c**) Transfection of miR-205-5p mimic into A549 and Calu-6 cell lines dramatically increased miR-205-5p levels (*P* = 0.0004 for A549, *P* < 0.0001 for Calu-6). (**d**) Overexpression of miR-205-5p led to a significant increase in TAp63 mRNA in both cell lines (*P* = 0.001 for A549, *P* = 0.0049 for Calu-6). (**e**) Western blot results showed that miR-205-5p overexpression increased TAp63 protein in the A549 cell line. Although qRT-PCR showed an increase in TAp63 mRNA in both cell lines, western blot results indicated that miR-205-5p expression decreased TAp63 protein in Calu-6 cells. The western blot images were cropped from the original images, which are available in the supplementary file. For each cell line, a separate SDS-PAGE gel was run. Each gel included a molecular weight marker, extracted protein from cells transfected with NC, and extracted protein from cells transfected with miR-205-mimic. After transferring the proteins from the SDS-PAGE gel to a PVDF membrane, the membrane was incubated with an anti-TP63 antibody. Following visualizing the bands, the membrane was washed and re-incubated with an anti-β-actin antibody. (**f**) In the A549 cell line, P21 (*P* = 0.0003) and BAX (*P* = 0.0049) were reduced, but DICER (*P* < 0.0001) elevated noticeably. (**g**) In the Calu-6 cell line, P21 (*P* < 0.0001), BAX (*P* < 0.0001), and DICER (*P* = 0.0035) were reduced at the same time. *P* value < 0.05: *, *P* value < 0.01: **, *P* value < 0.001: ***, *P* value < 0.0001: ****.

To investigate the direct interaction between miR-205-5p and the 5’ UTR of TAp63, we conducted a luciferase reporter assay. The data indicated that co-transfection of the miR-205-5p mimic with the pGL3-W plasmid significantly increased luciferase activity (*P* = 0.0103). In contrast, the miR-205-5p mimic with the pGL3-M plasmid did not affect luciferase activity. These findings suggest TAp63 is a direct target of miR-205-5p, and their interaction leads to an elevation in TAp63 expression (Fig. 5a, b). Then, we investigated the impact of miR-205-5p overexpression on the A549 and Calu-6 cell lines. qRT-PCR results demonstrated that transfecting the miR-205-5p mimic into A549 and Calu-6 cells resulted in an increase in TAp63 mRNA levels in both cell lines (Fig. 5c, d). Subsequent protein analysis using a Western blot assay revealed that overexpression of the miR-205-5p mimic elevated TAp63 protein levels in the A549 cell line; however, the protein levels decreased in Calu-6 cells (Fig. 5e). Additionally, we evaluated the impact of the miR-205-5p mimic on TAp63 downstream genes, including *P21*, *BAX*, and *DICER*. The results indicated a significant reduction in BAX and P21 expression in both cell lines. Interestingly, the expression patterns of DICER differed between the two cell lines: A549 cells exhibited an increase in DICER expression, while Calu-6 cells showed a decrease (Fig. 5f, g).

## miR-205-5p enhanced cell proliferation and inhibited migration in the A549 cell line

**Fig. 6:**
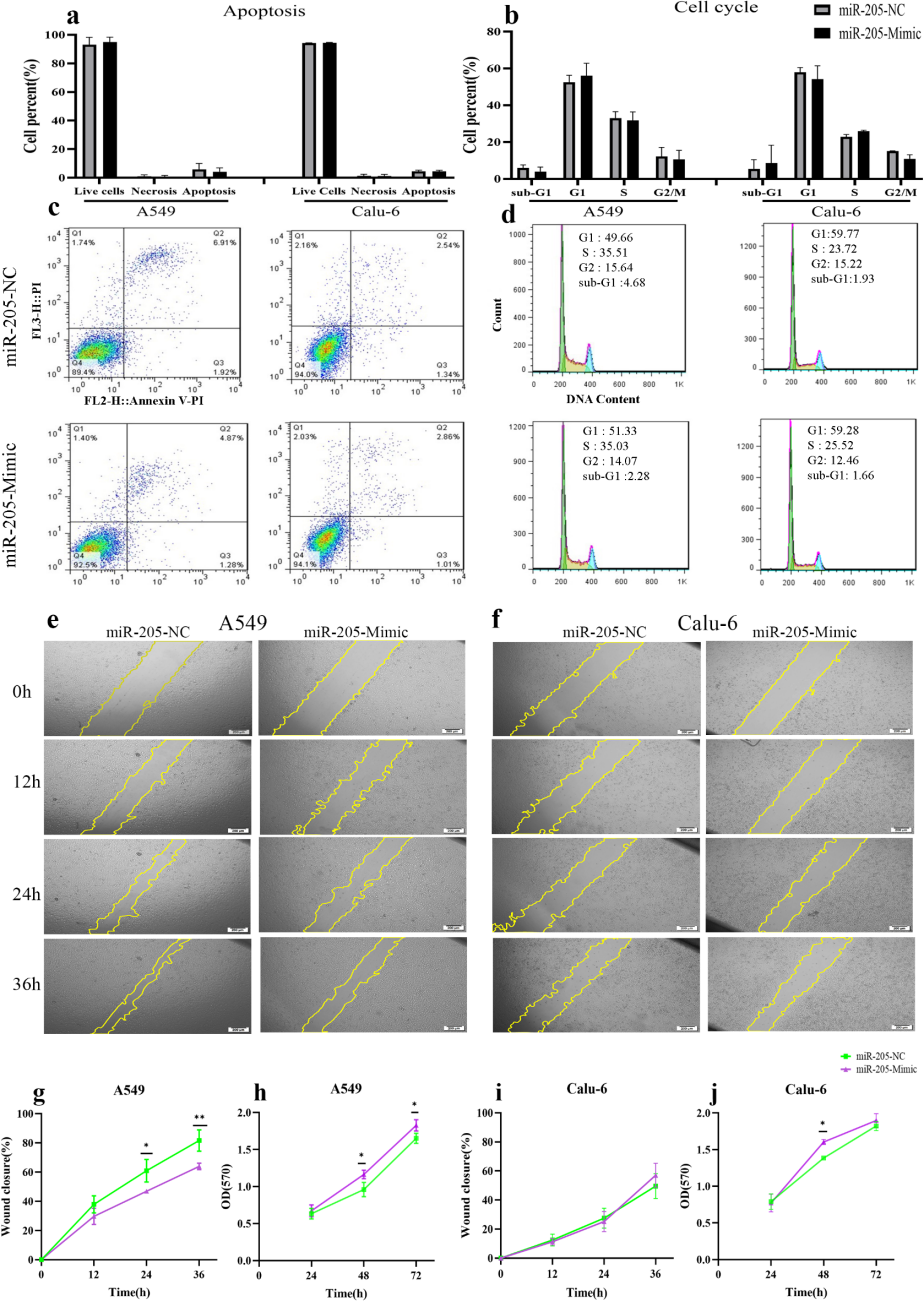
Implications of miR-205-5p overexpression on cellular activities. (**a**,** b**,** c**,** d**) Similar to ∆Np63α overexpression, transfecting miR-205-5p mimic had no significant effect on the apoptosis rate or cell cycle in both cell lines. (**e**,** g**) Results of the wound-healing assay showed that miR-205-5p overexpression substantially reduced migration in the A549 cell line (*P* = 0.0113 at 24 h, *P* = 0.0047 at 36 h). (**f**,** i**) miR-205-5p overexpression did not alter migration in Calu-6 cells. (**h**) In the A549 cell line, miR-205-5p overexpression significantly increased proliferation rates 48 h (*P* = 0.0353) and 72 h (*P* = 0.0393) after transfection. (**j**) In Calu-6 cells, miR-205-5p mimic transfection increased proliferation only 48 h after transfection (*P* = 0.0171). *P* value < 0.05: *, *P* value < 0.01: **.

Overexpression of miR-205-5p in the A549 and Calu-6 cell lines did not alter the rate of apoptosis or cell cycle, similar to what was observed after ΔNp63α overexpression (Fig. 6a, b, c, d). The results of the wound-healing assay in A549 cells were consistent with the effects of ΔNp63α overexpression on wound-healing. The data showed that overexpression of the miR-205-5p mimic significantly inhibited cell migration in the A549 cell line at 24 (*P* = 0.0113) and 36 h (*P* = 0.0047) after scratching (Fig. 6e, g). However, in Calu-6 cells, the miR-205-5p mimic did not impact migration (Fig. 6f, i). Results from the MTT assay indicated an increase in cell proliferation following miR-205-5p overexpression in both cell lines (Fig. 6h, j). It is worth noting that the effects of miR-205-5p on Calu-6 cell activities were not as pronounced as those observed in A549 cells.

## The long isoforms expressed in Calu-6 and A549 are not identical

**Fig. 7:**
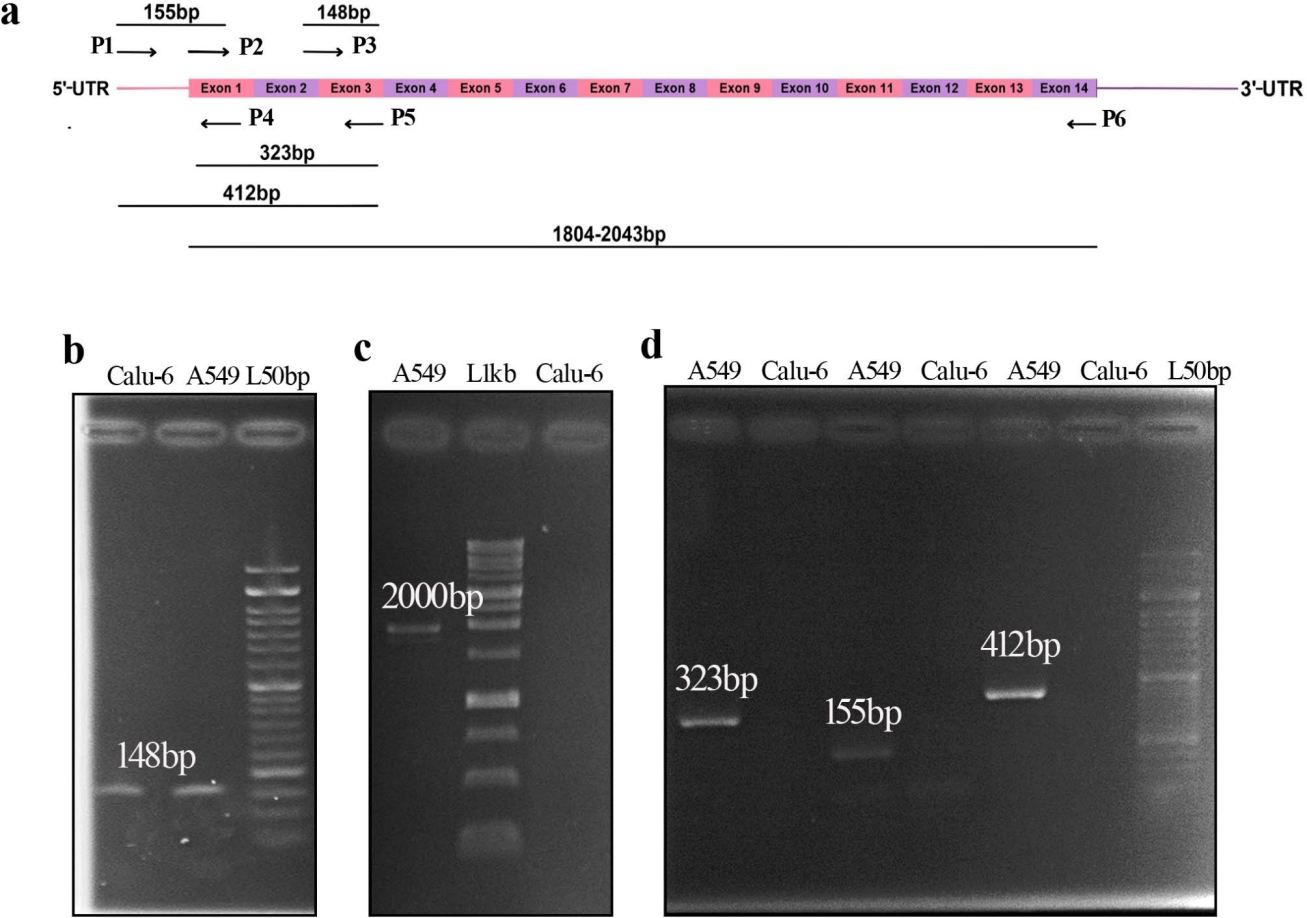
**The** ***TP63*** **isoform expressed in the Calu-6 cell line has a different 5’ region.** (**a**) The schematic diagram illustrates the TAp63α isoform and the site of the used primers. (**b**) PCR using P3 and P5 (specific qRT-PCR primers) led to *TP63* isoform amplification in both cell lines. (**c**) CDS-specific primers amplified a nearly 2000 bp fragment of the *TP63* isoform present in the A549 cell line. The *TP63* isoform expressed in the Calu-6 cell line was not amplified with these primers. (**d**) Only the *TP63* isoform expressed in the A549 cell line was amplified by primers for the exon1 region. These findings suggest that the *TP63* isoform in the Calu-6 cell line has a different 5’-UTR and lacks the miR-205-5p target site in its 5’-UTR. The agarose gel images were cropped from the original images, which are available in the supplementary file.

Due to the conflicting effects of miR-205-5p on TAp63 protein expression in A549 and Calu-6 cells, we decided to identify the expressed isoforms in these cell lines. After analyzing the amplified bands on agarose gel and considering Western blot results and a previous study^22^, we concluded that the expressed isoform in A549 cells is isoform 1 (TAp63α). Amplification using qRT-PCR primers (P3, P5) was performed in the Calu-6 cell line (Fig. 7b). However, amplification was not observed when using CDS-specific primers (Fig. 7c) or specific primers for exon 1 (Fig. 7d). Based on the evidence, it appears that the *TP63* isoform in Calu-6 has differences in the 5’-UTR and does not contain a target site for miR-205-5p.

## Discussion

Reports indicate that different *TP63* isoforms can act as either tumor suppressors or oncogenes^4^. Similarly, the function of miR-205 in cancer is controversial^12^. Due to this complexity, it is essential to thoroughly examine the role of *TP63* isoforms and miR-205 in each specific type of cancer.

In humans, miR-205 is encoded by the miR-205 host gene (*MIR205HG*), and its expression is controlled by transcriptional and post-transcriptional mechanisms^12^. Several studies have shown that TP63 regulates miR-205 expression at the transcriptional level^21,23,24^. According to Tran et al.‘s study, ΔNp63α increases the expression of miR-205 in bladder cancer cells, while ΔNp63α knockdown reduces the expression of miR-205 by decreasing RNA Pol II’s binding to the *MIR205HG*promoter^21^. Similar results have been observed in prostate cancer^23,24^.

In our study, we found a positive correlation between miR-205-5p and ΔNp63α expression in LUAD and LUSC tissue samples, consistent with our bioinformatics analysis. According to the bioinformatics analysis (Fig. 1b) and previous studies^25,26^, *TP63* isoforms are expressed in a small portion of LUAD samples. Therefore, the lack of a significant correlation between TAp63 and miR-205-5p in LUAD samples may be due to the small sample size.

qRT-PCR analysis showed that overexpression of ΔNp63α led to an increase in miR-205-5p levels in the A549 cell line. This result supports our co-expression study findings on datasets and tissue samples, indicating that ΔNp63α may function as a transcription factor for miR-205-5p in the A549 cell line.

In the A549 cell line, in addition to the upregulation miR-205-5p following ΔNp63α overexpression, we also observed an upregulation of TAp63α. We then evaluated the expression of *P21*, *BAX*, and *DICER* as downstream genes of TAp63s isoforms^4^. The qRT-PCR analysis revealed that while TAp63α was upregulated, P21 and BAX levels decreased. This contradiction might be due to ΔNp63α’s inhibitory effect on P21 and BAX expression^27,28^. Additionally, some studies have shown that miR-205-5p expression decreases P21 and BAX levels^29^. Therefore, both miR-205-5p and ΔNp63α prevent upregulation of P21 and BAX by TAp63α. In summary, it appears that ΔNp63α and TAp63α do not collaborate in regulating cell cycle and proliferation genes.

Consistent with previous studies^30,31^, we observed that ΔNp63α overexpression did not significantly affect apoptosis but increased proliferation in A549 and Calu-6 cells.

Additionally, we observed that ΔNp63α upregulated TAp63α and miR-205-5p, enhanced DICER and MET markers while decreasing EMT markers. Also, wound-healing assay data showed that ΔNp63α overexpression greatly inhibited migration in A549 cells. Previous studies have shown that ΔNp63α overexpression inhibits prostate and bladder cancer cell migration through miR-205^21,24^. It has also been found that TAp63 directly regulates DICER transcription and reduces metastasis^32^. Based on these findings, we speculated that ΔNp63α controls migration by regulating miR-205-5p, TAp63, and DICER expression. Our next step was to investigate the impact of miR-205-5p on TAp63 and DICER expression, as well as cellular activities.

The luciferase assay confirmed a direct interaction between miR-205-5p and the TAp63 5’-UTR, resulting in an increase in luciferase activity. Most studies have reported interactions between miRNAs and the 3’-UTR of mRNAs^33^. However, evidence suggests that interactions between miRNAs and the 5’-UTR can also regulate gene expression^34–37^. The majority of these studies indicate that microRNA binding to the 5’-UTR leads to enhanced target gene translation. For example, miR-10a positively influences global protein synthesis by binding to the 5’-UTR of mRNA encoding ribosomal proteins and stimulating translation^34^. It has also been reported that miR-346 increases APP translation and Aβ production by binding to the 5’-UTR of APP-related mRNA^35^. A study conducted by Panda et al. showed that miR-196b and HuD, known as repressors of translation, share the same target site in the 5’-UTR of the long insulin2 splice isoform, and miR-196b enhances translation by occupying the HuD target site^36^. The results of the luciferase assay, Western blot, and qRT-PCR indicate that miR-205-5p has a positive effect on both the translation and transcription of TAp63α in A549 cells. Future research should investigate the mechanism by which miR-205-5p affects TAp63 isoforms translation.

Similar to the observations after transfecting with ΔNp63α, we found that overexpression of miR-205-5p in A549 cells led to an increase in TAp63α and DICER levels. We also noted that miR-205-5p overexpression inhibited migration in this cell line, which is consistent with Zeng et al.^38^. Therefore, based on our data and previous studies, it appears that there is a ΔNp63α/miR-205-5p/TAp63α/DICER axis that regulates migration in A549 cell lines.

While initial studies suggested that short and long isoforms have opposing effects on cancer, some research has shown a potential interaction between them^39,40^. For instance, it has been reported that TAp63 knockout results in ΔNp63 silencing and triggers EMT events^39^. In fact, our study demonstrates a collaboration between short and long *TP63* isoforms in controlling migration in lung adenocarcinoma cells.

The upregulation of DICER may be linked to an increase in miRNAs that inhibit EMT.A study has indicated that in breast cancer, DICER decreases the expression of EMT markers and enhances the expression of MET markers by upregulating miR-200^41^. Additionally, it has been observed that miR-373 activates the E-Cadherin gene by interacting with its promoter^42^. Therefore, to clarify the mechanism by which ΔNp63α inhibits migration, it is suggested that the expression of these microRNAs be investigated in future studies.

Unlike the A549 cell line, in Calu-6 cells, overexpression of ΔNp63α decreased levels of miR-205-5p. It seems that the function of ΔNp63α on the *MIR205HG* gene promoter may be influenced by cellular context. Several studies have shown that a transcription factor can bind to different sites and its role can change from activator to repressor in different biological contexts. Interactions with other transcription factors, cofactors, or epigenetic modifications that affect DNA accessibility could be responsible for this contextual effect^43^.

Furthermore, after transfecting Calu-6 cells with the miR-205-5p mimic, we observed an increase in TAp63 at the mRNA level but a decrease at the protein level. Nam et al. concluded that the presence of different 3’-UTR isoforms could explain the variability in cellular response to a microRNA^44^. Our data indicated that the TAp63 isoform expressed in Calu-6 cells is different from that in A549 cells and does not contain a binding site for miR-205-5p. This difference may account for the varied results observed in Calu-6 cells compared to A549 cells when overexpressing ΔNp63α and miR-205-5p. These findings suggest that when studying microRNA interactions with 5’-UTRs, it is important to consider 5’-UTR isoforms.

Reports on the impact of miR-205 in in vitro assays on lung adenocarcinoma cell lines are inconsistent. Some studies suggest that miR-205 inhibits apoptosis^45,46^, while others indicate that miR-205 has no effect on apoptosis^29^, which aligns with our findings. In our study, we observed that ΔNp63α and miR-205-5p did not significantly impact apoptosis in A549 and Calu-6 cells. However, in line with previous research on miR-205’s effects on NSCLC^29,45–47^, we discovered that miR-205-5p increased proliferation in both cell lines.

Contrary to common belief, our observations in A549 cells show that overexpression of ΔNp63α or miR-205-5p has opposite effects on proliferation and migration. This consistent with some studies that suggest proliferation and migration can occur in different directions^48,49^. For example, miR-146b has been shown to increase proliferation but hinder migration and invasion^48^. Additionally, Bui et al. found oscillatory expression of ΔNp63^50^, noting that depletion of ΔNp63 in primary breast adenocarcinoma tumors initiates migration and invasion while inhibiting proliferation.

**Fig. 8:**
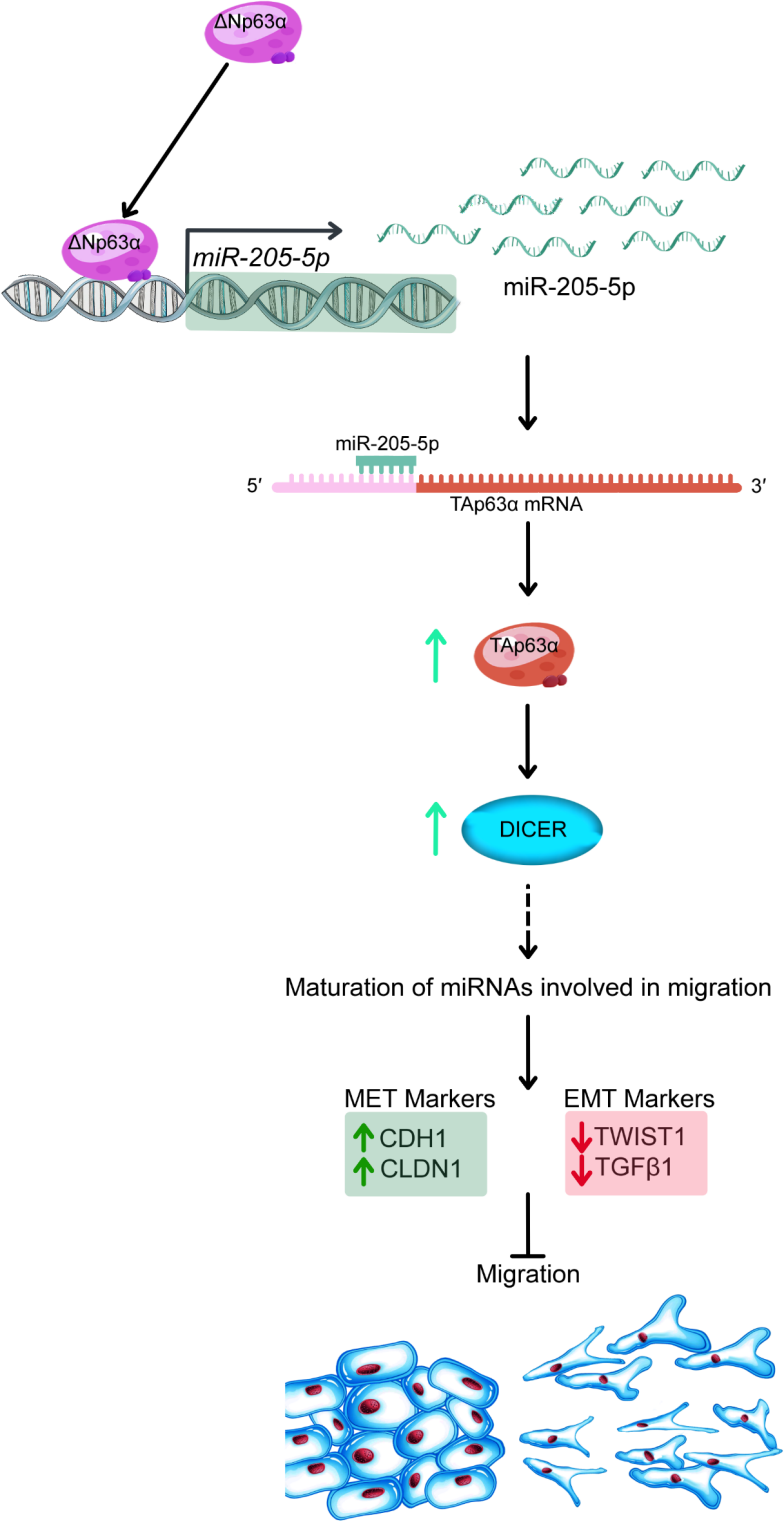
Summary of Results. ΔNp63α functions as a transcription factor for MIR205HG, promoting the expression of miR-205-5p. miR-205-5p, in turn, targets the 5’-UTR of the TAp63α isoform, leading to its upregulation. Elevated TAp63α levels enhance the expression of DICER, which modulates the biogenesis of multiple microRNAs (not analyzed in this study). These molecular changes contribute to increased expression of MET markers and reduced expression of EMT markers. These events ultimately inhibit cell migration in lung adenocarcinoma cells.

Our overall results suggest that ΔNp63α possibly acts as a transcription factor for miR-205-5p in the A549 cell line. Additionally, it appears that in A549 cells, ΔNp63α regulates TAp63α expression in part through miR-205-5p. Based on our findings, we believe that the ΔNp63α/miR-205-5p/TAp63α/DICER axis could be one of the potential pathways that regulates migration in lung adenocarcinoma (Fig. 8). Furthermore, we discovered that microRNAs could lead to the up-regulation of a tumor suppressor, which may open the door for microRNA therapeutic applications. Considering our observations in Calu-6 cells, it is essential to take into account tumor content when utilizing the ΔNp63α/miR-205-5p/TAp63α/DICER axis or miR-205-5p in therapeutic applications.

## Methods

### In silico analysis

The RNA-seq and miRNA-seq data for LUAD, LUSC, and non-tumor tissues were downloaded from the Cancer Genome Atlas (TCGA) database. Among the TAp63 isoforms, TAp63α was selected for in silico analysis due to its identification as the major isoform of TAp63s in previous studies^22^. A co-expression study was conducted using the R program, with a *P* value < 0.05 considered statistically significant.

## Tissue samples and RNA extraction

Lung cancer tissues were sourced from the National Tumor Bank of Iran, as described previously^51,52^. Briefly, all samples were immediately frozen in liquid nitrogen upon collection and subsequently stored at −80 °C until RNA extraction. All biopsies were obtained prior to the initiation of any specific drug treatments or chemotherapy. The study protocol was reviewed and approved by the Ethics Committee of Tarbiat Modares University (Approval Code: IR.MODARES.REC.1400.082). Total RNA were extracted from homogenized tissues by One Step-RNA reagent (Thermo Fisher Scientific, USA). After assessing the RNA quality, a total of 14 adenocarcinoma samples and 7 squamous cell carcinoma samples, meeting quality standards were selected.

## Cell culture

All cell lines were obtained from the Pasteur Institute of Iran (Tehran, Iran). HEK293T, A549, and KYSE cells were cultured in Dulbecco’s Modified Eagle Medium (DMEM) with high glucose levels (Gibco™, USA), while Calu-6 cells were cultured in Roswell Park Memorial Institute (RPMI) medium (Gibco™, USA). Both culture media were supplemented with 10% fetal bovine serum (FBS) (Gibco™, USA). Cells were incubated in a humid incubator with 5% CO2 at 37 °C.

### Plasmid construction

The KYSE cell line cDNA was used as a PCR template to amplify of ∆Np63α. The ∆Np63α fragment was generated using coding sequence (CDS)-specific primers, which included HindIII and XhoI restriction sites (Thermo Fisher Scientific, USA). The amplified fragment was then cloned into the pcDNA 3.1 (+) vector (Invitrogen, USA), resulting in the development of the pcDNA-∆Np63 construct. For the luciferase assay, the wild-type (W) or mutated (M) sequence of human TAp63 5’-UTR was amplified using specific primers with NcoI and HindIII restriction sites (Thermo Fisher Scientific, USA). These amplified products were subsequently cloned into a pGL3-control vector (Promega, USA). Table 1 includes a list of primer sequences used in this study.

### Transfection, RNA extraction, cDNA synthesis, and qRT-PCR

Transfections were carried out using Lipofectamine 3000 Transfection Reagent (Invitrogen, USA) following the manufacturer’s instructions. 16–20 h before transfection, 4 × 10^4^/well of A549 and 6 × 10^4^/well of Calu-6 cells were seeded in 24-well plates. After the incubation period, 40 nM of either miR-205-5p mimic or miR-205-5p negative control (NC) (Genepharma, China) were transfected into the cells. For plasmid transfection, 500 ng of pcDNA-∆Np63 or pcDNA-Mock were used. 48 h post-transfection, total RNA was extracted using the GeneAll^®^ RiboEx™ solution (GeneAll, South Korea). DNase treatment was performed using DNase I (Thermo Fisher Scientific, USA) on the extracted RNA. cDNA synthesis was carried out using the BioFact™ cDNA Synthesis Kit (BioFact, India). For the quantification of miR-205-5p in cell lines, polyadenylation was performed using the *E. coli* Poly (A) Polymerase kit (NEB, USA) prior to cDNA synthesis. The U6 gene served as the reference for miR-205-5p quantification, while GAPDH was used as the reference gene for other target genes. In tissue samples, the expression of miR-205-5p was assessed using the stem-loop method, with the 5S gene as the internal control. For evaluating the expression of ΔNp63 and TAp63, the ACTB gene was used as the internal reference. qRT-PCR was performed using RealQ Plus 2x Master Mix Green (Amplicon, Denmark). Table 1 provides the specific primers used for quantifying all genes.

### Luciferase assay

A luciferase assay was performed to evaluate the direct interaction between miR-205-5p and the TAp63 5’-UTR. HEK293T cells were cultured in a 48-well plate. The following day, 100 ng of each plasmid and 60 nM of either miR-205-5p mimic or NC were transfected into the cells using Lipofectamine 3000. 48 hours’ post-transfection, luciferase activity was measured using the ONE-Step™ Luciferase Assay System (BPS Bioscience, USA). The measured luciferase activity was normalized against the total protein content.

### MTT assay

Sixteen to twenty-four hours before transfection, 4 × 10^3^ A549 cells and 6 × 10^3^ Calu-6 cells were seeded in 96-well plates. Transfection was performed using Lipofectamine 3000. The assay was conducted at 24-, 48-, and 72- hours post-transfection, with three replicates for each experiment. At each time point, 10 µL of MTT reagent (from a 5 mg/mL stock solution) (Sigma-Aldrich, USA) was added to each well, followed by incubation for 4 h at 37 °C. Subsequently, the MTT solution was carefully removed, and dimethyl sulfoxide (DMSO) (Merck, Germany) was added to dissolve the formazan crystals. The absorbance was measured at 570 nm using a spectrophotometer to assess cell viability.

### Apoptosis

miR-205-5p mimic/NC or pcDNA-ΔNp63/Mock were transfected into A549 and Calu-6 cell lines. Forty-eight hours post-transfection, the cells were harvested and stained with the Annexin-V-FLUOS Staining Kit (Roche, Switzerland) and then incubated for 15 min at room temperature in a dark environment. Subsequently, the apoptosis rate was quantified using the FACS Calibur Flow Cytometry System. Data analysis was done using FlowJo software version 10.

### Cell cycle assay

Forty-eight hours after transfection of miR-205-5p mimic/NC or pcDNA-ΔNp63/Mock, A549 and Calu-6 cells were harvested and fixed. The fixed cells were then stained with propidium iodide and incubated for 30 min in a dark environment at room temperature. The DNA content within the stained cells was determined using the FACSCalibur Flow Cytometry System. The data were analyzed using FlowJo software version 10.

### Western blot

48 h after transfection, cells were harvested, and 1 × 10^6^ cells were lysed using a lysis buffer. In the following steps, the Bradford assay was used to quantify protein concentration. The proteins were then separated on an SDS-PAGE gel and transferred to a PVDF membrane using a standard transfer buffer. The membrane was then immersed in a blocking buffer containing a 2% non-fat dry milk solution for 75 min at room temperature. A primary antibody incubation was carried out overnight (β-Actin (2A3): sc-517582, Dicer (F-10): sc-136979, p63 (D-9): sc-25268; all from Santa Cruz Biotechnology, USA). This step was followed by a 75-minute incubation with secondary antibodies: m-IgGκ BP-HRP and mouse anti-rabbit IgG-HRP (Santa Cruz Biotechnology, USA). The protein bands were visualized using Pierce ECL Western Blotting Substrate (Thermo Fisher Scientific, USA), and the band intensity was analyzed using IMAGE J software.

### Wound healing assay

Wound-healing assay was performed to assess the impact of miR-205-5p or ΔNp63α on cell migration. 5 × 10^4^ A549 cells and 8 × 10^4^ Calu-6 cells were placed in a 24-well plate. After 16–24 h, the cells were transfected with miR-205-5p mimic/NC or pcDNA-ΔNp63/Mock. 12–24 h after transfection, at nearly 100% confluency, a 200-µl pipette tip was used to scratch the cells. Afterwards, the cells were then rinsed twice with 1x PBS, and the media was replaced with fresh media containing 1% fetal bovine serum. This was done to support minimal growth conditions. Cell migration was monitored and captured using an inverted microscope at various intervals. The percentage of wound closure was measured using ImageJ software.

### **Characterization of*TP63*** e**xpressed isoforms**

For the characterization of expressed isoforms, we used specific primers for CDS and exon1 (including 5’-UTR and a part of coding sequence) of TAp63α isoform (Fig. 7a). Specific primers for CDS and exon1 were used for the characterization of expressed isoforms of TAp63. CDS-specific primers were applied to amplify long isoforms 1, 2, 7, and 10. Isoforms 3 and 13 were not amplified with these primers. Excluding isoforms 3 and 13 from the study did not affect the results, as isoform 3’s molecular weight did not match the western blot results, and isoform 13 lacks the miR-205-5p target site. The primer sequences are listed in Table 1.

### Statistical analysis

The 2^-(ΔΔCt)^ method was utilized for gene expression comparisons between the two groups (pcDNA -Mock/ pcDNA-∆Np63, NC/Mimic) and ΔCt was used for correlation studies of tissue samples. All experiments were analyzed using GraphPad Prism 9 (GraphPad Software, San Diego, California, USA, www.graphpad.com). The mean and standard deviations were calculated for each parameter between the two groups. *P* values were analyzed using Student’s t-test and one-way ANOVA, with statistical significance was considered at *P* < 0.05.

**Table 1:** Sequences of primers and oligonucleotides used in this study.

Primer or oligonucleotide name	Sequence (5’>3’)
TAp63 5’-UTR-Wild-F (P1)	CCCAAGCTTCCCGGCTTTATATC
TAp63 5’-UTR-Wild -R	CATGCCATGGTGGCTTCCTTCAACTGTC
TAp63 5’-UTR-Mutant -F	CCCAAGCTTCCCGGCTTTATATC
TAp63 5’-UTR-Mutant -R	CATGCCATGGTGGCAACTGTCTTTGATATCAACG
TAp63-Exon1-R (P4)	TCACCGCTGGATGTAAG
TAp63-CDS-F (P2)	CCCAAGCTTATGAATTTTGAAACTTCACGGTG
TAp63-CDS-R (P6)	CC CTCGAG TCACTCCCCCTCCTCTTTGATG
ΔNp63α-CDS-F	CCCAAGCTTATGTTGTACCTGGAAAACAATGC
ΔNp63α-CDS-R	CC CTCGAG TCACTCCCCCTCCTCTTTGATG
TAp63-F (P3)	GGGATTTTCTGGAACAGCCT
TAp63-R (P5)	CACATGGGGTCACTCAGGTC
ΔNp63-F	CCAGACTCAATTTAGTGAGCCAC
ΔNp63-R	GGGTGATGGAGAGAGAGCATC
GAPDH-F	ATGGGGAAGGTGAAGGTCG
GAPDH-R	GGGGTCATTGATGGCAACAA
ACTB-F	TACAGGAAGTCCCTTGCCATC
ACTB-R	CTATCACCTCCCCTGTGTGG
BAX-F	TCAGGATGCGTCCACCAAGAAG
BAX-R	TGTGTCCACGGCGGCAATCATC
P21-F	CCTGTCACTGTCTTGTACCCTTG
P21-R	GCGTTTGGAGTGGTAGAAATCT
DICER-F	CACATGGCTGAGAAGTATACCTGTC
DICER-R	AAAATTGTCCATCATGTCCTCGC
CDH1 -F	GCTCTTTGACCACCGCTCTC
CDH1 -R	CGTGTGTGACTGTGAAGGGG
CLDN1-F	TTGGGCTTCATTCTCGCCTT
CLDN1-R	TTGCTTGCAATGTGCTGCT
TWIST1-F	GGAGTCCGCAGTCTTACGAG
TWIST1-R	TCTGGAGGACCTGGTAGAGG
TGFβ1-F	CAATTCCTGGCGATACCTCAG
TGFβ1-R	AACCACTGCCGCACAACT
Anchored Oligo(dt)-A	CCAGTGAGCAGAGTGACGAGGACTCGAGCTCAAGCTTTTTTTTTTTTTTTTA
Anchored Oligo(dt)-C	CCAGTGAGCAGAGTGACGAGGACTCGAGCTCAAGCTTTTTTTTTTTTTTTTC
miR-205-5p-F	CCATTGTCCTTCATTCCACCG
miR-205-5p stem-loop	GTCGTATCCAGTGCAGGGTCCGAGGTATTCGCACTGGATACGACCAGACT
Universal reverse primer for stem-loop	CCAGTGCAGGGTCCGAGGTA
5 S-F	GTCTACGGCCATACCACCCTG
5 S-R	AAAGCCTACAGCACCCGGTAT
U6-F	GAACGATACAGAGAAGATTAGC
Q-Outer	CCAGTGAGCAGAGTGACG
miR-205-5p mimic	UCCUUCAUUCCACCGGAGUCUG
miR-205-5p NC	UUGUACUACACAAAAGUACUG

## Electronic supplementary material

Below is the link to the electronic supplementary material.

Supplementary Material 1

## Data Availability

All data generated or analyzed during this study are included in this published article and its supplementary information file.

## Abbreviations

TP63: Tumor protein 63
EMT: Epithelial-mesenchymal transition
MET: Mesenchymal-epithelial transition
TAD: Transcriptional activation domain
LUSC: Lung squamous cell carcinoma
LUAD: Lung adenocarcinoma
NSCLC: Non-small cell lung cancer
miRNA: microRNA
miR-205: microRNA-205
TCGA: The cancer genome atlas
DMEM: Dulbecco’s modified eagle medium
RPMI: Roswell park memorial institute
FBS: Fetal bovine serum
CDS: Coding sequence
NC: Negative control
DMSO: Dimethyl sulfoxide
MIR205HG: miR-205 host gene

## References

[1] Leiter, A., Veluswamy, R. R. & Wisnivesky, J. P. The global burden of lung cancer: current status and future trends. *Nat. Reviews Clin. Oncol.* **20** (9), 624–639 (2023). 10.1038/s41571-023-00798-3 37479810

[2] Qian, J. & Massion, P. P. Role of chromosome 3q amplification in lung cancer. *J. Thorac. Oncol.* **3** (3), 212–215 (2008). 18317062 10.1097/JTO.0b013e3181663544

[3] Li, Y. et al. p63: a crucial player in epithelial stemness regulation. *Oncogene* **42** (46), 3371–3384 (2023). 37848625 10.1038/s41388-023-02859-4 PMC10638092

[4] Chen, Y. et al. A double dealing Tale of p63: an oncogene or a tumor suppressor. *Cell. Mol. Life Sci.* **75**, 965–973 (2018). 28975366 10.1007/s00018-017-2666-y PMC11105324

[5] Xu, Y., Yang, X., Xiong, Q., Han, J. & Zhu, Q. The dual role of p63 in cancer. *Front. Oncol.* **13**, 1116061 (2023). 37182132 10.3389/fonc.2023.1116061 PMC10174455

[6] Samanta, A., Saha, P., Johnson, O., Bishayee, A. & Sinha, D. Dysregulation of delta Np63 alpha in squamous cell carcinoma and its therapeutic targeting. Biochimica et Biophysica Acta (BBA)-Reviews on Cancer. :189034. (2023). 10.1016/j.bbcan.2023.189034 38040268

[7] Pokorná, Z., Vysloužil, J., Hrabal, V. & Vojtěšek Bi, Coates, P. J. The foggy world (s) of p63 isoform regulation in normal cells and cancer. *J. Pathol.* **254** (4), 454–473 (2021). 33638205 10.1002/path.5656

[8] Napoli, M. et al. Genome-wide p63-target gene analyses reveal TAp63/NRF2-dependent oxidative stress responses. *Cancer Res. Commun.* **4** (2), 264–278 (2024). 38165157 10.1158/2767-9764.CRC-23-0358 PMC10832605

[9] Massion, P. P. et al. Significance of p63 amplification and overexpression in lung cancer development and prognosis. *Cancer Res.* **63** (21), 7113–7121 (2003). 14612504

[10] Iacono, M. L. et al. p63 and p73 isoform expression in non-small cell lung cancer and corresponding morphological normal lung tissue. *J. Thorac. Oncol.* **6** (3), 473–481 (2011). 21289519 10.1097/JTO.0b013e31820b86b0

[11] Abolfathi, H., Arabi, M. & Sheikhpour, M. A literature review of MicroRNA and gene signaling pathways involved in the apoptosis pathway of lung cancer. *Respir. Res.* **24** (1), 55 (2023). 36800962 10.1186/s12931-023-02366-w PMC9938615

[12] Ferrari, E. & Gandellini, P. Unveiling the ups and downs of miR-205 in physiology and cancer: transcriptional and post-transcriptional mechanisms. *Cell Death Dis.* **11** (11), 980 (2020). 33191398 10.1038/s41419-020-03192-4 PMC7667162

[13] Chauhan, N., Manojkumar, A., Jaggi, M., Chauhan, S. C. & Yallapu, M. M. microRNA-205 in prostate cancer: overview to clinical translation. Biochimica et biophysica acta (BBA)-Reviews on cancer. ;**1877**(6):188809. (2022). 10.1016/j.bbcan.2022.188809 PMC9996811 36191828

[14] Plantamura, I., Cataldo, A., Cosentino, G. & Iorio, M. V. miR-205 in breast cancer: state of the Art. *Int. J. Mol. Sci.* **22** (1), 27 (2020). 33375067 10.3390/ijms22010027 PMC7792793

[15] La Ferlita, A. et al. Non-coding RNAs in endometrial physiopathology. *Int. J. Mol. Sci.* **19** (7), 2120 (2018). 30037059 10.3390/ijms19072120 PMC6073439

[16] Tian, Y. et al. MiRNAs in radiotherapy resistance of nasopharyngeal carcinoma. *J. Cancer*. **11** (13), 3976 (2020). 32328201 10.7150/jca.42734 PMC7171507

[17] He, L. et al. Ovarian cancer cell-secreted Exosomal miR-205 promotes metastasis by inducing angiogenesis. *Theranostics* **9** (26), 8206 (2019). 31754391 10.7150/thno.37455 PMC6857047

[18] Charkiewicz, R. et al. Validation for histology-driven diagnosis in non‐small cell lung cancer using hsa‐mi R‐205 and hsa‐mi R‐21 expression by two different normalization strategies. *Int. J. Cancer*. **138** (3), 689–697 (2016). 26311306 10.1002/ijc.29816

[19] Jiang, M. et al. Relative expressions of miR-205-5p, miR-205-3p, and miR-21 in tissues and serum of non-small cell lung cancer patients. *Mol. Cell. Biochem.* **383**, 67–75 (2013). 23881177 10.1007/s11010-013-1755-y

[20] Hezova, R. et al. MiR-205 functions as a tumor suppressor in adenocarcinoma and an oncogene in squamous cell carcinoma of esophagus. *Tumor Biology*. **37**, 8007–8018 (2016). 26711784 10.1007/s13277-015-4656-8

[21] Tran, M. N. et al. The p63 protein isoform ∆Np63α inhibits epithelial-mesenchymal transition in human bladder cancer cells: role of MIR-205. *J. Biol. Chem.* **288** (5), 3275–3288 (2013). 23239884 10.1074/jbc.M112.408104 PMC3561548

[22] Sethi, I. et al. A global analysis of the complex landscape of isoforms and regulatory networks of p63 in human cells and tissues. *BMC Genom.* **16**, 1–15 (2015). 10.1186/s12864-015-1793-9 PMC4528692 26251276

[23] Gandellini, P. et al. miR-205 regulates basement membrane deposition in human prostate: implications for cancer development. *Cell. Death Differ.* **19** (11), 1750–1760 (2012). 22555458 10.1038/cdd.2012.56 PMC3469086

[24] Tucci, P. et al. Loss of p63 and its microRNA-205 target results in enhanced cell migration and metastasis in prostate cancer. *Proc. Natl. Acad. Sci.* **109** (38), 15312–15317 (2012). 22949650 10.1073/pnas.1110977109 PMC3458363

[25] Au, N. et al. P63 expression in lung carcinoma: a tissue microarray study of 408 cases. *Appl. Immunohistochem. Mol. Morphology*. **12** (3), 240–247 (2004). 10.1097/00129039-200409000-00010 15551738

[26] Yaman, B., NArT, D., EKrEN, P. K., Çok, G. & VErAL, A. Expression of p63, TTF-1 and Maspin in non-small cell lung carcinoma and their effect on the prognosis and differential diagnosis. *Turk. Patoloji Derg*. **31** (3), 163–174 (2015). 26456962 10.5146/tjpath.2015.01305

[27] Kudo, K. et al. ∆Np63α transcriptionally represses p53 target genes involved in the radiation-induced DNA damage response: ∆Np63α May cause genomic instability in epithelial stem cells. *Radiat. Oncol.* **17** (1), 183 (2022). 36380314 10.1186/s13014-022-02139-7 PMC9667649

[28] Chatterjee, A. et al. Regulation of p53 family member isoform ∆Np63α by the nuclear factor-κB targeting kinase IκB kinase Β. *Cancer Res.* **70** (4), 1419–1429 (2010). 20145131 10.1158/0008-5472.CAN-09-2613 PMC2963198

[29] Zhao, Y. L. et al. MiR-205‐5p promotes lung cancer progression and is valuable for the diagnosis of lung cancer. *Thorac. Cancer*. **13** (6), 832–843 (2022). 35076182 10.1111/1759-7714.14331 PMC8930496

[30] Yoshida, M. et al. Development of an integrated CRISPRi targeting ∆Np63 for treatment of squamous cell carcinoma. *Oncotarget* **9** (49), 29220 (2018). 30018747 10.18632/oncotarget.25678 PMC6044376

[31] Wu, J. et al. ∆Np63α activates CD82 metastasis suppressor to inhibit cancer cell invasion. *Cell Death Dis.* **5** (6), e1280–e (2014). 24901051 10.1038/cddis.2014.239 PMC4611714

[32] Su, X. et al. TAp63 suppresses metastasis through coordinate regulation of Dicer and MiRNAs. *Nature* **467** (7318), 986–990 (2010). 20962848 10.1038/nature09459 PMC3055799

[33] Ha, M. & Kim, V. N. Regulation of MicroRNA biogenesis. *Nat. Rev. Mol. Cell Biol.* **15** (8), 509–524 (2014). 25027649 10.1038/nrm3838

[34] Ørom, U. A., Nielsen, F. C. & Lund, A. H. MicroRNA-10a binds the 5′ UTR of ribosomal protein mRNAs and enhances their translation. *Mol. Cell*. **30** (4), 460–471 (2008). 18498749 10.1016/j.molcel.2008.05.001

[35] Long, J. M., Maloney, B., Rogers, J. T. & Lahiri, D. K. Novel upregulation of amyloid-β precursor protein (APP) by microRNA-346 via targeting of APP mRNA 5′-untranslated region: implications in Alzheimer’s disease. *Mol. Psychiatry*. **24** (3), 345–363 (2019). 30470799 10.1038/s41380-018-0266-3 PMC6514885

[36] Panda, A. C. et al. miR-196b-mediated translation regulation of mouse insulin2 via the 5′ UTR. *PLoS One*. **9** (7), e101084 (2014). 25003985 10.1371/journal.pone.0101084 PMC4086887

[37] Lytle, J. R., Yario, T. A. & Steitz, J. A. Target mRNAs are repressed as efficiently by microRNA-binding sites in the 5′ UTR as in the 3′ UTR. *Proc. Natl. Acad. Sci.* **104** (23), 9667–9672 (2007). 17535905 10.1073/pnas.0703820104 PMC1887587

[38] Zeng, Y. et al. Repression of Smad4 by miR–205 moderates TGF-β-induced epithelial-mesenchymal transition in A549 cell lines Corrigendum in/10.3892/ijo. 5166. International journal of oncology. 2016;49(2):700-8. (2021). 10.3892/ijo.2016.3547 27279345

[39] Katoh, I., Tsukinoki, K., Hata, R-I. & Kurata, S. ∆Np63 silencing, DNA methylation shifts, and epithelial-mesenchymal transition resulted from TAp63 genome editing in squamous cell carcinoma. *Neoplasia* **45**, 100938 (2023). 37778252 10.1016/j.neo.2023.100938 PMC10544079

[40] Li, N. et al. TA-p63-γ regulates expression of ∆N-p63 in a manner that is sensitive to p53. *Oncogene* **25** (16), 2349–2359 (2006). 16331262 10.1038/sj.onc.1209270

[41] Hsu, T-W. et al. Dicer-mediated miR-200b expression contributes to cell migratory/invasive abilities and cancer stem cells properties of breast cancer cells. *Aging (Albany NY)*. **14** (16), 6520 (2022). 35951366 10.18632/aging.204205 PMC9467414

[42] Place, R. F., Li, L-C., Pookot, D., Noonan, E. J. & Dahiya, R. MicroRNA-373 induces expression of genes with complementary promoter sequences. Proceedings of the National Academy of Sciences. ;105(5):1608-13. (2008). 10.1073/pnas.0707594105 PMC2234192 18227514

[43] Weidemüller, P., Kholmatov, M., Petsalaki, E. & Zaugg, J. B. Transcription factors: Bridge between cell signaling and gene regulation. *Proteomics* **21** (23–24), 2000034 (2021). 10.1002/pmic.202000034 34314098

[44] Nam, J-W. et al. Global analyses of the effect of different cellular contexts on MicroRNA targeting. *Mol. Cell*. **53** (6), 1031–1043 (2014). 24631284 10.1016/j.molcel.2014.02.013 PMC4062300

[45] Jiang, M. et al. Reduced expression of miR–205–5p promotes apoptosis and inhibits proliferation and invasion in lung cancer A549 cells by upregulation of ZEB2 and downregulation of erbB3. *Mol. Med. Rep.* **15** (5), 3231–3238 (2017). 28350117 10.3892/mmr.2017.6398

[46] Duan, B. et al. miR-205 as a biological marker in non-small cell lung cancer. *Biomed. Pharmacother.* **91**, 823–830 (2017). 28501009 10.1016/j.biopha.2017.04.086

[47] Cai, J. et al. miR-205 targets PTEN and PHLPP2 to augment AKT signaling and drive malignant phenotypes in non–small cell lung cancer. *Cancer Res.* **73** (17), 5402–5415 (2013). 23856247 10.1158/0008-5472.CAN-13-0297

[48] Yan, M. et al. miR-146b promotes cell proliferation and increases chemosensitivity, but attenuates cell migration and invasion via FBXL10 in ovarian cancer. *Cell Death Dis.* **9** (11), 1123 (2018). 30409964 10.1038/s41419-018-1093-9 PMC6224598

[49] Evdokimova, V., Tognon, C., Ng, T. & Sorensen, P. H. Reduced proliferation and enhanced migration: two sides of the same coin? Molecular mechanisms of metastatic progression by YB-1. *Cell. Cycle*. **8** (18), 2901–2906 (2009). 19713745 10.4161/cc.8.18.9537

[50] Bui, N. H. et al. Spatiotemporal regulation of ∆Np63 by TGFβ-regulated MiRNAs is essential for cancer metastasis. *Cancer Res.* **80** (13), 2833–2847 (2020). 32312834 10.1158/0008-5472.CAN-19-2733 PMC7385751

[51] Roknabadi, N., Borghei, Y-S., Seifezadeh, S. S., Soltani, B. M. & Mowla, S. J. Selective Naked-Eye detection of lung squamous cell carcinoma mediated by LncRNA SOX2OT targeted nanoplasmonic probe. *ACS Omega*. **9** (35), 37205–37212 (2024). 39246497 10.1021/acsomega.4c04565 PMC11375807

[52] Seifzadeh, S. S., Borghei, Y-S., Roknabadi, N. & Mowla, S. J. A novel approach of differentiation of adenoma and carcinoma in lung cancer based on biogenic in situ synthesis of gold nanostructures on various oligonucleotide motifs. *Microchim. Acta*. **191** (11), 1–10 (2024). 10.1007/s00604-024-06744-z 39438316

