# Supplementary material for: The interaction between ΔNp63α and TAp63α, mediated by miR-205-5p, inhibits the migration of lung adenocarcinoma cells
Source: Sci Rep. 2025 Apr 3;15:11501. doi: 10.1038/s41598-025-95206-4 (PMC11968970; doi:10.1038/s41598-025-95206-4)
Supplement: Supplementary file 1 — Supplementary Material 1 [file 41598_2025_95206_MOESM1_ESM.pdf]

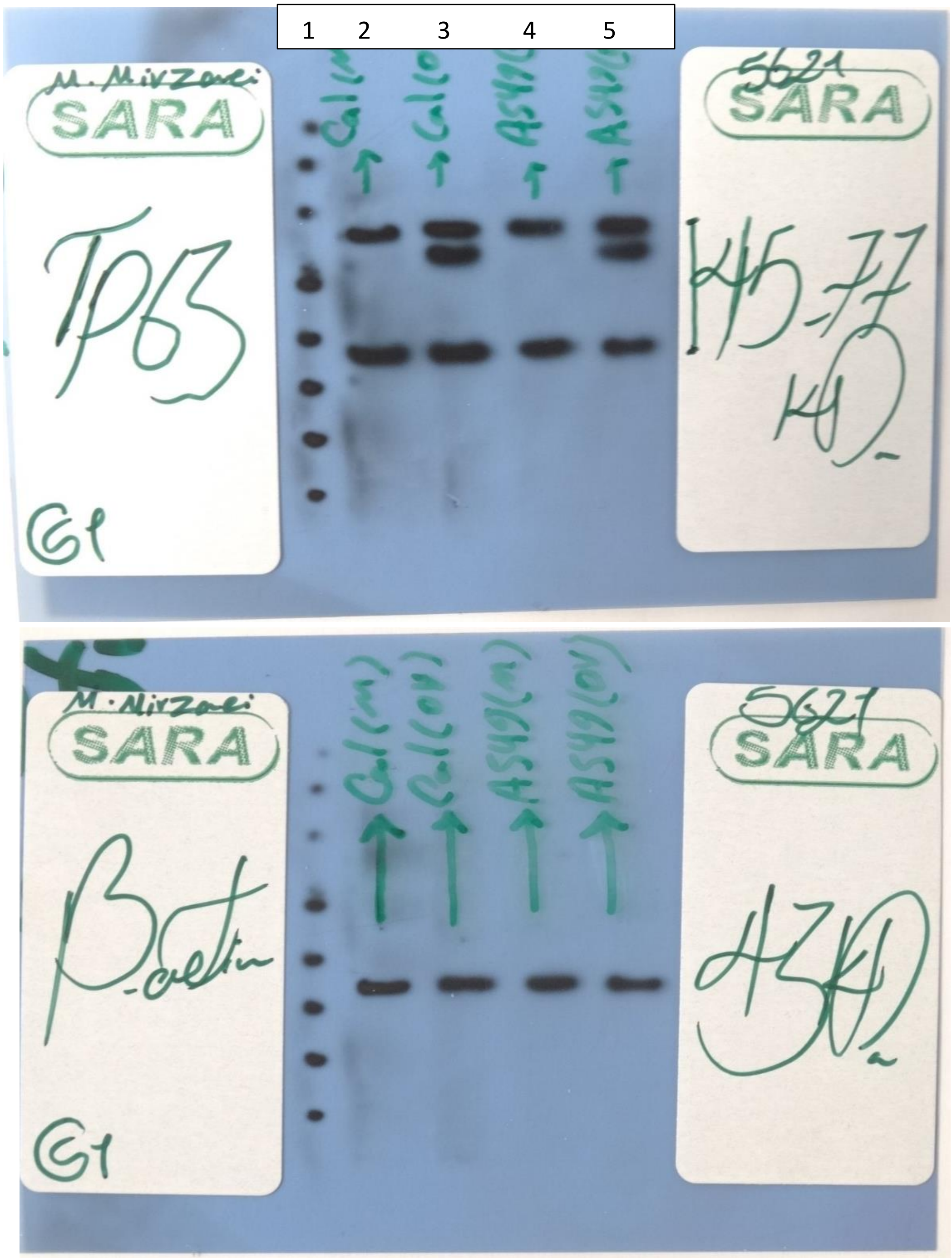

Figure 3c, f: Western blot analysis of TP63 expression following ΔNp63α overexpression in Calu-6 and A549 cells. Western blot images showing the expression of TP63 isoforms in Calu-6 and A549 cell lines. Lane 1: Molecular weight marker, Lane 2: Calu-6 cells transfected with pcDNA-Mock, Lane 3: ΔNp63α overexpression in Calu-6 cells, Lane 4: A549 cells transfected with pcDNA-Mock, Lane 5: ΔNp63α overexpression in A549 cells. Results indicate that TAp63 expression increases in both cell lines upon ΔNp63α overexpression.

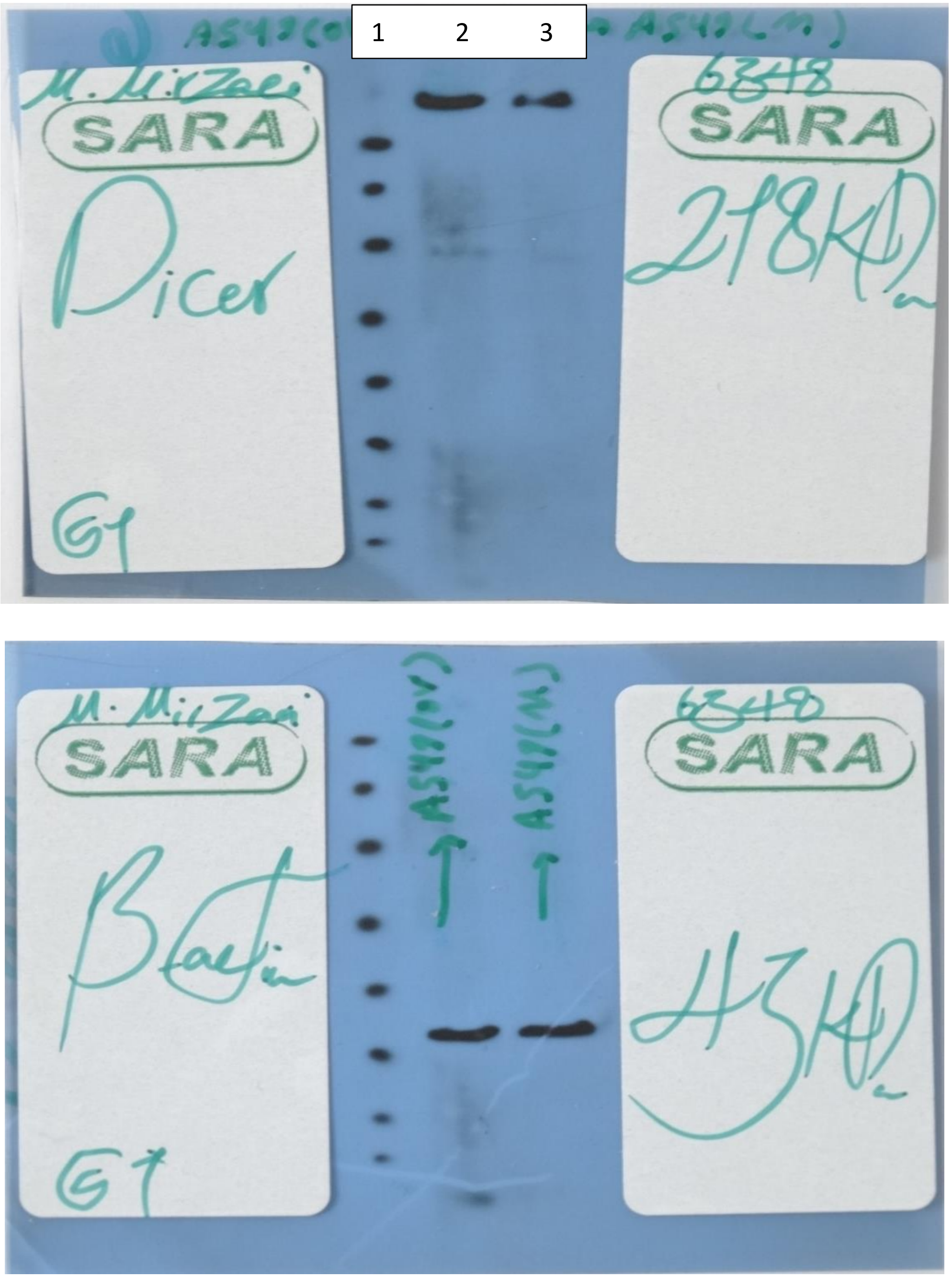

Figure 3g: Western blot analysis of DICER expression following ΔNp63α overexpression in A549 cells. Western blot images showing the expression of DICER in A549 cells. Lane 1: Molecular weight marker, Lane 2: ΔNp63α overexpression in A549 cells, Lane 3: pcDNA-Mock in A549 cells. Results indicate that ΔNp63α overexpression leads to increased expression of DICER.

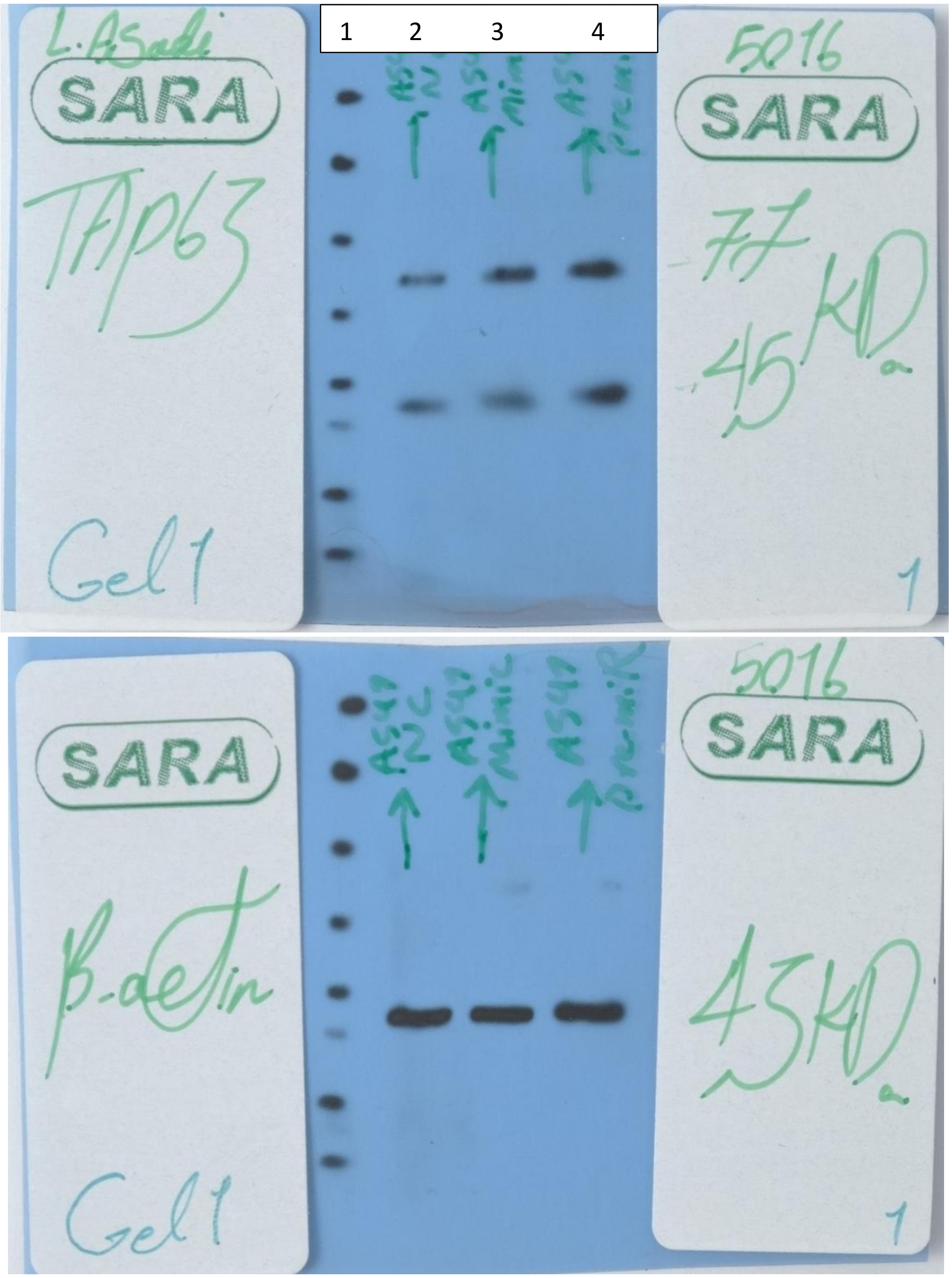

Figure 5e: Western blot analysis of TAp63 expression in A549 cells transfected with miR-205-5p mimic and negative control. Western blot images showing the expression of TAp63 in A549 cells. Lane 1: Molecular weight marker, Lane 2: A549 cells transfected with a negative control, Lane 3: A549 cells transfected with miR-205-5p mimic, Lane 4: A549 cells transfected with a pre-miR-205-expressing vector (not used for analysis in this study). Results demonstrate that transfection with miR-205-5p mimic leads to increased expression of TAp63.

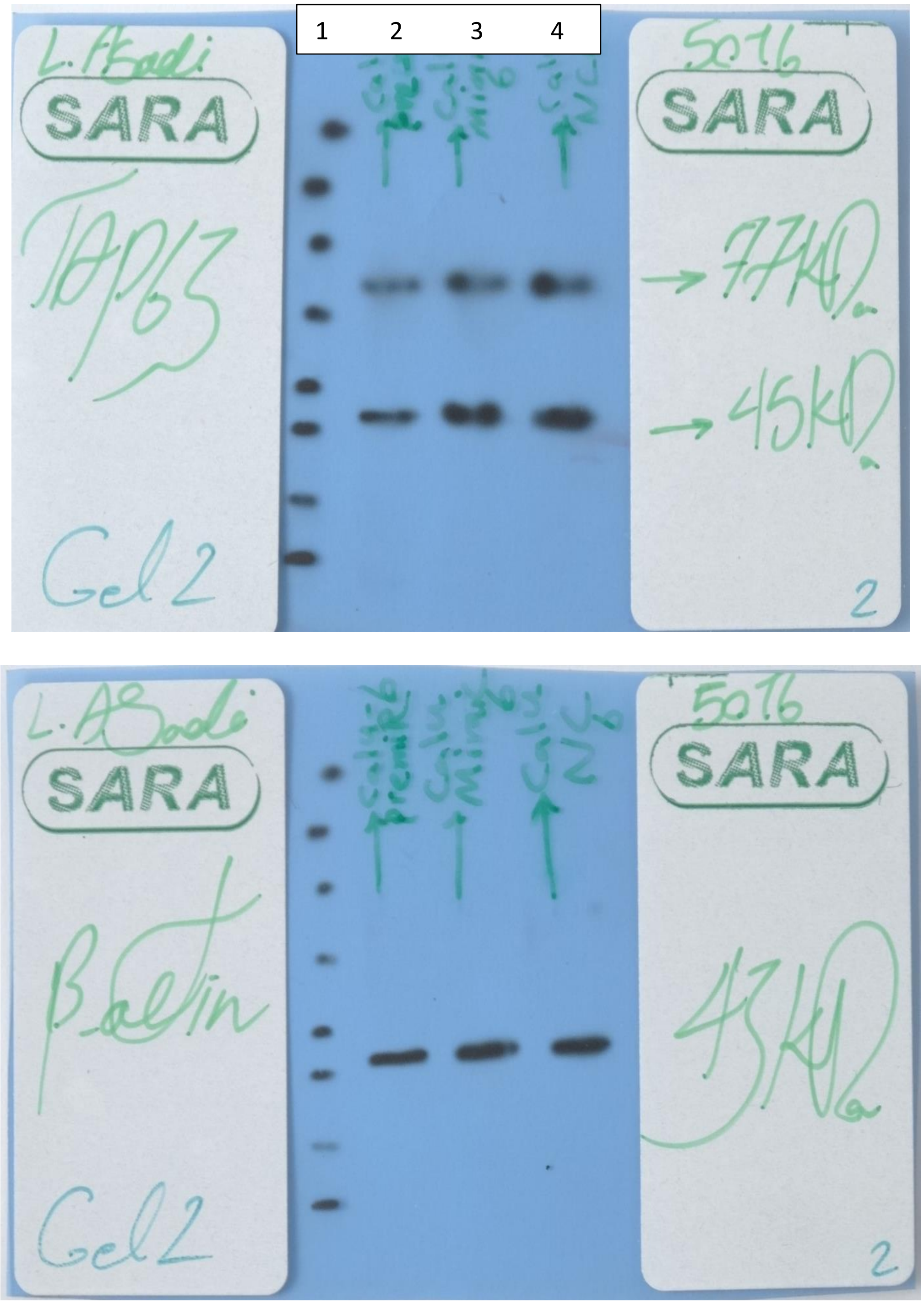

Figure 5e: Western blot analysis of TAp63 expression in Calu-6 cells transfected with miR-205-5p mimic and negative control. The western blot results demonstrate the effect of miR-205-5p mimic on the expression of TAp63 in Calu-6 cells. Lane 1: Molecular weight marker, Lane 2: Calu-6 cells transfected with a pre-miR-205-expressing vector (not used for analysis in this study), Lane 3: Calu-6 cells transfected with miR-205-5p mimic, Lane 4: Calu-6 cells transfected with a negative control. Results indicate that transfection with miR-205-5p mimic leads to a reduction in TAp63 expression.

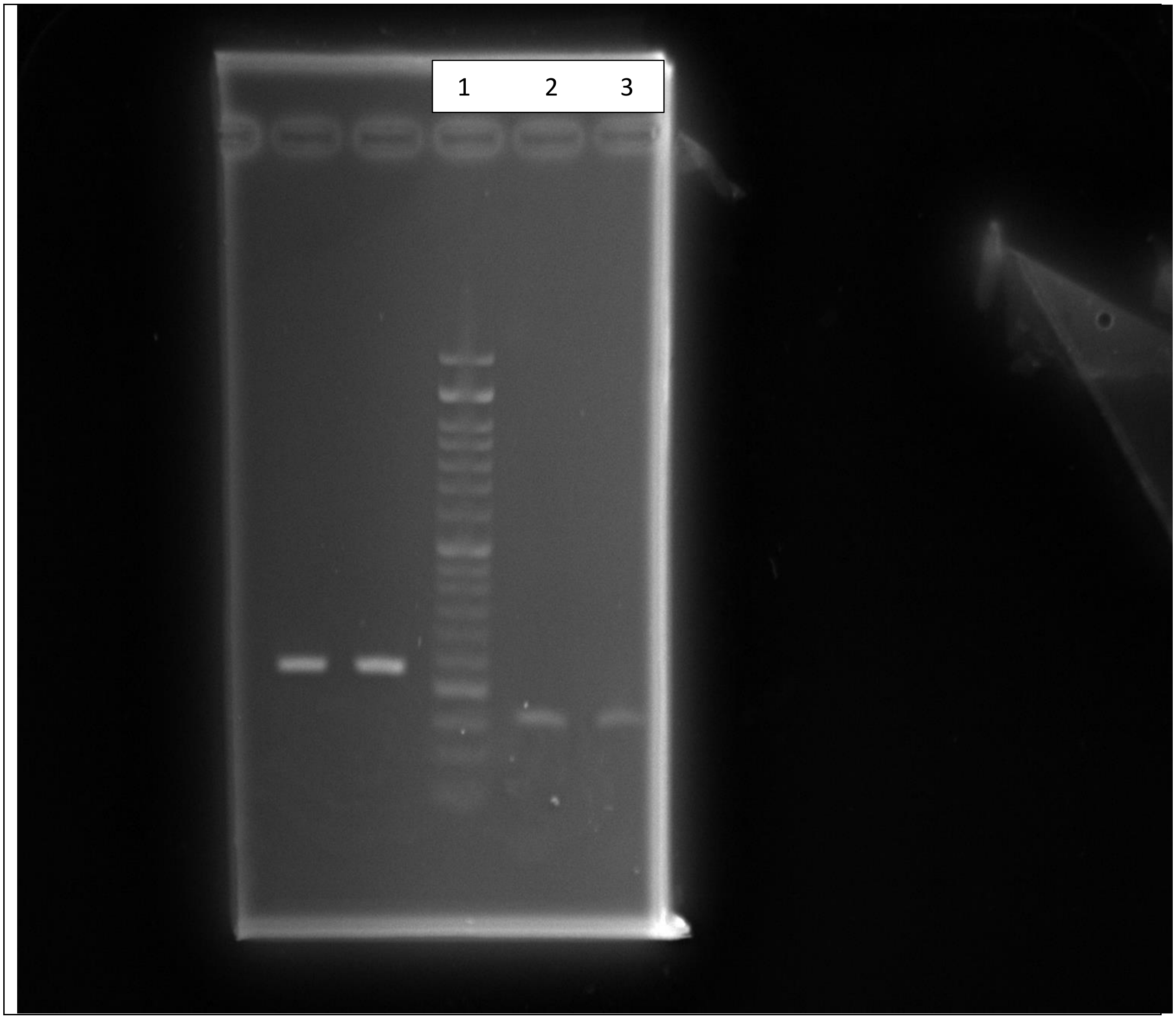

Figure 7b: Agarose gel of 148bp fragment (using specific real-time PCR primers). Lane1:50bp DNA ladder. Lane 2: Amplified fragment using A549 cDNA.Lane 3: Amplified fragment using Calu-6 cDNA. The results of the other lanes were not used in this study.

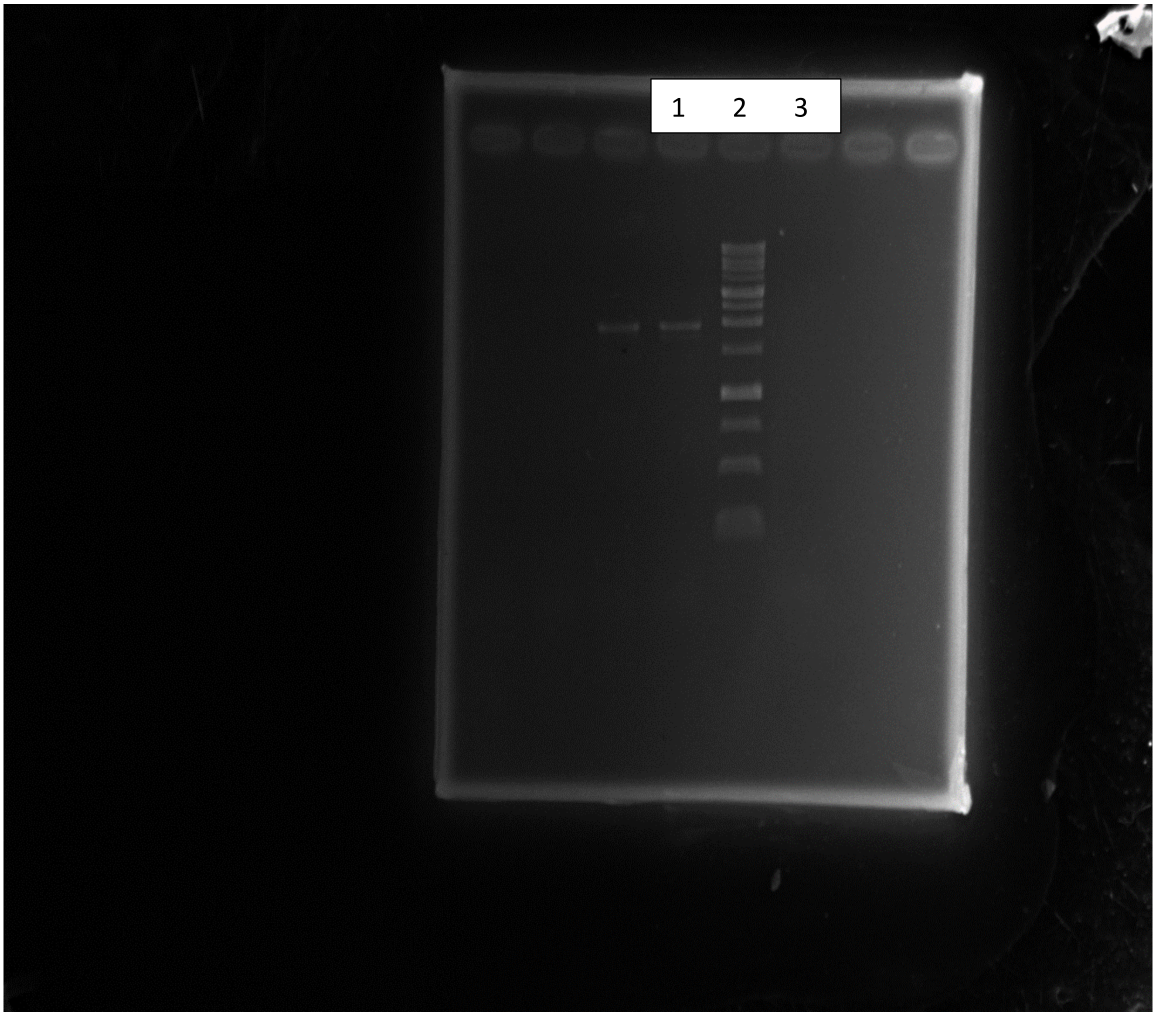

Figure 7c: Agarose gel of 2000bp fragment (amplified using CDS-specific primers). Lane 1: Amplified fragment using A549 cDNA.Lane 2: 1kb DNA ladder. Lane 3: Amplified fragment using Calu-6 cDNA. The results of the other lanes were not used in this study.

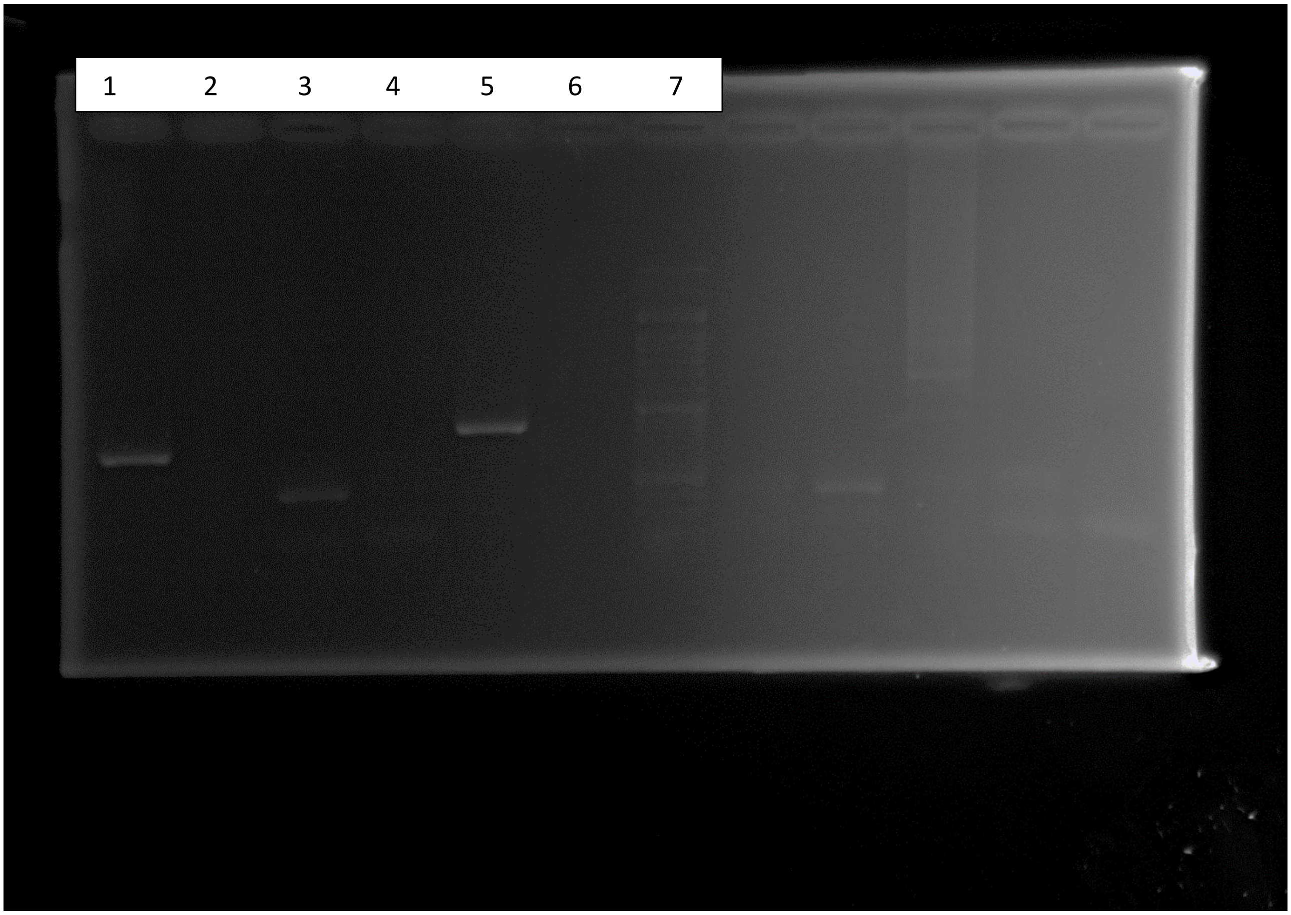

Figure 7d: Agarose gel of 323bp, 155bp, and 412bp fragments (amplified using exon1-specific primers). Lane 1, 3, 5: Amplified fragment using A549 cDNA.Lane 2, 4, 6: Amplified fragment using Calu-6 cDNA.Lane 7: 50bp DNA ladder. The results of the other lanes were not used in this study.
